# The effects of di-butyl phthalate exposure from medications on human sperm RNA among men

**DOI:** 10.1038/s41598-019-48441-5

**Published:** 2019-08-27

**Authors:** Molly Estill, Russ Hauser, Feiby L. Nassan, Alan Moss, Stephen A. Krawetz

**Affiliations:** 1 Center for Molecular Medicine and Genetics, Wayne State University School of Medicine, Detroit, MI 48201 USA; 2 Department of Obstetrics and Gynecology, Wayne State University School of Medicine, Detroit, MI 48201 USA; 3 Vincent Memorial Obstetrics and Gynecology Service, Massachusetts General Hospital, Harvard Medical School, and Departments of Environmental Health and Epidemiology, Harvard T.H. Chan School of Public Health, Boston, MA 02115 USA; 4 Departments of Environmental Health and Nutrition, Harvard T. H. Chan School of Public Health, MA, 02115 USA; 5 Department of Gastroenterology, Beth Israel Deaconess Medical Center, Boston, MA 02115 USA

**Keywords:** Environmental impact, Risk factors

## Abstract

Endocrine disruptors, such as phthalates, are suspected of affecting reproductive function. The Mesalamine and Reproductive Health Study (MARS) was designed to address the physiological effect of *in vivo* phthalate exposure on male reproduction in patients with Inflammatory Bowel Disease (IBD). As part of this effort, the effect on sperm RNAs to DBP exposure were longitudinally assessed using a cross-over cross-back binary design of high or background, exposures to DBP. As the DBP level was altered, numerous sperm RNA elements (REs) were differentially expressed, suggesting that exposure to or removal from high DBP produces effects that require longer than one spermatogenic cycle to resolve. In comparison, small RNAs were minimally affected by DBP exposure. While initial study medication (high or background) implicates different biological pathways, initiation on the high-DBP condition activated oxidative stress and DNA damage pathways. The negative correlation of REs with specific genomic repeats suggests a regulatory role. Using ejaculated sperm, this work provides insight into the male germline’s response to phthalate exposure.

## Introduction

Humans are ubiquitously exposed to, exogenous chemicals that act as endocrine disruptors. There are many different types of phthalates and their metabolites have varied capacities for modifying the endocrine system^1–3^. They are known to interact through peroxisome proliferator‐activated receptors (PPAR) and xenobiotic sensors^1,2^. Ortho-phthalates are commonly used as solvents and plasticizers in numerous consumer products, such as personal care products and polyvinyl chloride plastics^4^. They have also been incorporated into the coatings of some medications^5–9^. Although considerable literature suggests that gestational and neonatal phthalate exposure is detrimental to reproductive function of the offspring^10^, the health effects of phthalates on human male reproductive function is not fully understood. Epidemiological studies of adult phthalate exposures and semen parameters suggest that some phthalates are associated with abnormal sperm morphology^11^, sperm concentration^12,13^, oxidative stress^14^, and DNA damage^15^. Among couples undergoing IVF, urinary phthalate concentrations in the male partner are inversely correlated with high-quality blastocysts^14^, suggesting a male effect. While the intergenerational impact of phthalate exposures remain unknown, murine intergenerational models, and limited human data, suggests that paternal experiences, can have phenotypic consequences in the offspring^16–22^. This intergenerational effect is suspected of being mediated through epigenetic mechanisms, in part by RNAs and/or chromatin and its modifiers delivered by the spermatozoon at fertilization^16,19–21,23^. The impact of phthalates are beginning to be assessed^14,24,25^.

The Mesalamine and Reproductive Health Study (MARS) (NCT01331551) was initiated (https://clinicaltrials.gov/ct2/show/NCT01331551) to directly address the *in vivo* effect of phthalate exposure on male reproduction. The MARS study was designed to assess semen quality^5^ and hormone levels^6,7^ in human males through a novel cross-over and cross-back study after exposure to, and removal of exposure to very high-DBP. Using a crossover design and longitudinal data structure, each subject acted as their own control, thus mitigating the genetic and environmental variation that often complicates causal assessment. Patients with Inflammatory Bowel Disease (IBD) are often prescribed mesalamine, a medication which, in some formulations, contains di-butyl phthalate (DBP) in the coating to allow for release of the active ingredient in the distal small intestine and colon^9,26^. The DBP-coated medication(s), at maximal dosages, range from 300–700% of the designated EPA Reference Dose (RfD) for a 150-pound individual^27–29^. The use of the DBP-coated medication produces urinary monobutyl-phthalate (MBP) levels 1000x higher than the levels found in the general U.S. population^5^. The MARS study recruited 73 individuals, who provided up to six semen, urine, and blood samples. As described in detail^5^, a subset of the subjects provided longitudinal samples across alternating high and background DBP exposures, with 60 subjects enrolled in the full protocol^5^.

Ejaculated spermatozoa, and their RNAs provide a snapshot of transcriptomic processes, capturing the influence of the paternal environment^16,19,20,30^ during spermatogenesis. The current study applied RNA-seq to the MARS sperm samples, generating both a series of long RNA (>200 nucleotides) and small (<200 bp) RNA libraries to elucidate the biological processes being modified through phthalate exposure. The transcriptomic effects of both IBD and DBP exposure were assessed as a function of sperm RNA elements (REs), to provide a robust quantitative measure of effect^31^. Differential responses to high-DBP exposures were readily apparent in DBP-naïve men and men chronically exposed to high-DBP mesalamine. RNA levels of transcribed genomic repeats were examined to determine which genomic repeats were well-represented in human sperm and which genomic repeats were part of the response to high-DBP mesalamine.

## Results

**Figure 1:**
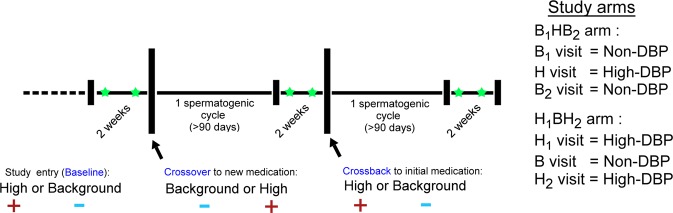
Crossover study design. Men enter the study having taken a mesalamine medication coated with (+) or without (−) DBP for at least 3 months. Semen, blood, and urine were collected twice (green star) at baseline, after 4 months on an alternate drug (crossover), and after a final 4 months on the original drug (crossback).

The MARS study was designed to longitudinally assess semen quality in human males as a function of high-DBP exposures. The study design is summarized in Fig. 1. Subjects entered the study having taken mesalamine, an aminosalicylate, with or without a DBP containing coating for at least 3 months^5,32^. Asacol® and Asacol® HD’s active ingredient is mesalamine, that uses DBP as an excipient in the enteric coating, while numerous other mesalamine formulations, such as Pentasa®, Lialda®, or Apriso®, do not use DBP^5–7^. Semen, blood and urine were collected at baseline, after 3 months on the alternate drug (crossover), and after a final 3 months on the arm-specific baseline drug (crossback). In order to ensure that the ejaculated spermatozoa originate from a single medication exposure, a minimum duration of 90 days (~1 spermatogenic cycle) on each medication was required prior to sample collection. This ensured that for a given sample, an entire spermatogenic cycle and subsequent ductal transport would have occurred^33^ on the same medication.

In the B_1_HB_2_ arm, men entered the study on the non-DBP mesalamine i.e., background DBP exposure (B_1_ visit), transitioned to a high-DBP mesalamine (H visit), then returned to the non-DBP mesalamine (B_2_ visit). In the H_1_BH_2_ arm, subjects entered the study on high-DBP mesalamine (H_1_ visit), transitioned to a non-DBP mesalamine (B visit), then returned to the high-DBP mesalamine (H_2_ visit). Sperm and their corresponding RNAs were isolated, sequenced and quality assessed to remove failed samples (Supplementary Data). A total of 150 samples were available for analysis. In the H_1_BH_2_ arm and the B_1_HB_2_ arm, respectively, 19 and 16 subjects presented with consecutive visit pairs (e.g. baseline and crossover sets, or crossover and crossback sets) or visit trios (complete baseline, crossover, and crossback sets), with the remaining as bookended (e.g. baseline and crossback) or singletons.

### Sperm REs from IBD subjects are similar to control subjects

Several studies have applied RNA-seq to IBD with a primary focus on intestinal biopsies, not distal organs. Using the MARS B_1_HB_2_ study arm as an IBD cohort, the differences between sperm samples from control males from an independent study and the B_1_HB_2_ study samples were determined. The control dataset was composed of males from idiopathic infertile couples who produced a live birth after the use of assisted reproduction technologies. The veracity of the control dataset derived from Jodar *et al*, is known^34^. Similarly, the high-quality MARS samples (Supplementary Table S1) excluded somatic cells prior to RNA extraction, and exhibited RNA profiles similar to human testis. This was confirmed through comparison of the most abundant sperm RNA elements (REs)^31^ to transcripts known to be highly expressed in testis (Supplementary Table S2)^35^. The use of REs, rather than whole genes, permitted the fine resolution and detection of differential exons and intergenic regions^31^. This is of particular importance in spermatozoa, which can exhibit extensive RNA fragmentation^36^, compounded by alternative splicing.

**Figure 2:**
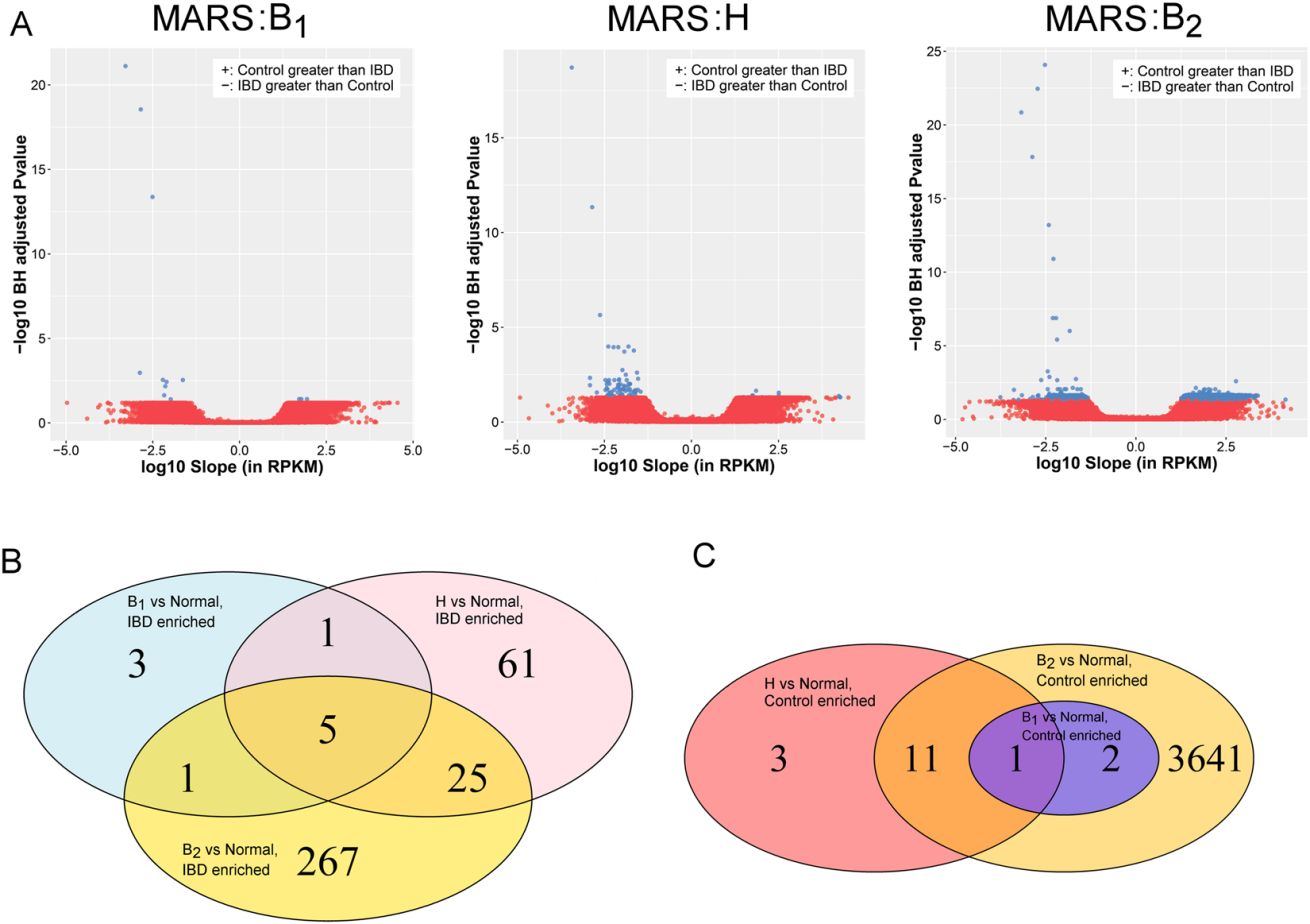
Normal and IBD differential REs. (**A**) Volcano plots of linear model results for all REs. Red points represent REs that are not statistically significant, while the relatively few blue points represent REs that have a Benjamini-Hochberg adjusted P-value less than 0.05 and an absolute slope of at least 10 RPKM. REs with a positive slope exhibit higher expression in control subjects, while those with a negative slope have higher level in IBD subjects. (**B**) Venn diagram of the REs enriched in the IBD subjects, across the three study visits. (**C**) Venn diagram of the REs enriched in the control subjects, across the three study visits.

To identify sperm RNAs altered by IBD, a linear model employing random resampling (to account for visit replicates) was applied to healthy controls and each study visit (i.e, baseline, crossover and crossback) of the B_1_HB_2_ study arm, producing three sets of differential REs (IBD baseline vs control, IBD crossover vs control, and IBD crossback vs control). To ensure confidence in the differential REs, only those REs altered in two or more comparisons were considered. Relatively few REs (40 REs) were altered in at least two study visit comparisons to healthy controls (Fig. 2). Only 14 REs were upregulated in controls and 26 REs were upregulated in IBD, the majority of which were exonic REs (Supplementary Table S3). The corresponding gene names of the 40 sperm REs did not overlap with several previous studies in either human or mouse colon biopsy microarrays or RNA-seq^37–45^, reflecting the vastly different tissue types. This suggests that the IBD condition or chronic exposure to the drug mesalamine, a nonsteroidal anti-inflammatory aminosalicylate, alone does not substantially alter the transcript profile of ejaculated spermatozoa. However, among the REs upregulated in IBD compared to controls, ANKRD36 and CDHR2 that map with high resolution to two or more differential exonic REs. Within the controls, TJP1 and ARPC1A yielded two or more differential up-regulated exonic REs. This suggests that select exons are consistently up-regulated or down-regulated in sperm from DBP-naïve subjects as compared to controls. Interestingly, both CDHR2, a non-classical cadherin, and TJP1, a tight junction adaptor protein, are associated with cell-cell interactions, suggesting that IBD may modify these interactions^46,47^. As TJP1 is involved in linking tight junction transmembrane proteins^47^, the observed reduction in TJP1 levels in IBD-afflicted individuals suggests that the testis’ tight junctions may also be adversely affected.

While the only cell type assessed in this study was spermatozoa, any phthalate associated effects on spermatozoa are likely to be mediated through other supporting cell types of the male reproductive system, e.g., Sertoli or other support cells, during spermatogenesis and/or spermiogenesis. Therefore, the effects observed in spermatozoa are likely downstream of the effected cell type. In support of the above we have noted that ANKRD36, TJP1, and ARPC1A (Supplementary Table S3**)** are expressed (containing at least 100 reads) in cultured human Sertoli cells^48^. In accord with the above this suggests that the effects of IBD on spermatozoa are mediated through Sertoli cells, although the effect of IBD during epididymal transit cannot be excluded.

### DBP-induced alterations in sperm RNA profiles

To assess the impact of high-DBP exposure on sperm RNAs, Linear Mixed-Effects Models (LMEM) were applied to the individual study arms, identifying the changes occurring during the baseline-crossover transition and the crossover-crossback transition. The final model adjusted for sequencing batch, subject’s time on high-DBP drug (prior to study initiation), subject’s Body Mass Index, seasonal warmth, subject’s smoking history, subject’s age, library amplification efficiency, duplication rate, and genomic alignment rate, while allowing the model’s intercept to vary across each subject. The implementation of a LMEM allowed for RNA changes across the predictive variable (i.e. study visit, indicating the use of high-DBP mediation or non-DBP mesalamine) to be assessed in each individual subject, while considering biological replicates. In each study arm and comparison, over three thousand REs were identified as differentially expressed (Supplementary Table S4**)**, when requiring an empirical P-value less than 0.05 and minimum absolute change of 10 RPKM. As summarized in Supplementary Table S4, during the H_1_ to B transition in the H_1_BH_2_ arm, a total of 1150 and 293 REs respectively were upregulated and downregulated, and 832 and 779 REs, respectively, upregulated and downregulated during the B to H_2_ transition. In the B_1_HB_2_ arm, 1021 and 2630 REs were upregulated and downregulated, respectively, during the B_1_ to H transition, and 665 and 666 REs were upregulated and downregulated, respectively, during the H to B_2_ transition.

**Figure 3:**
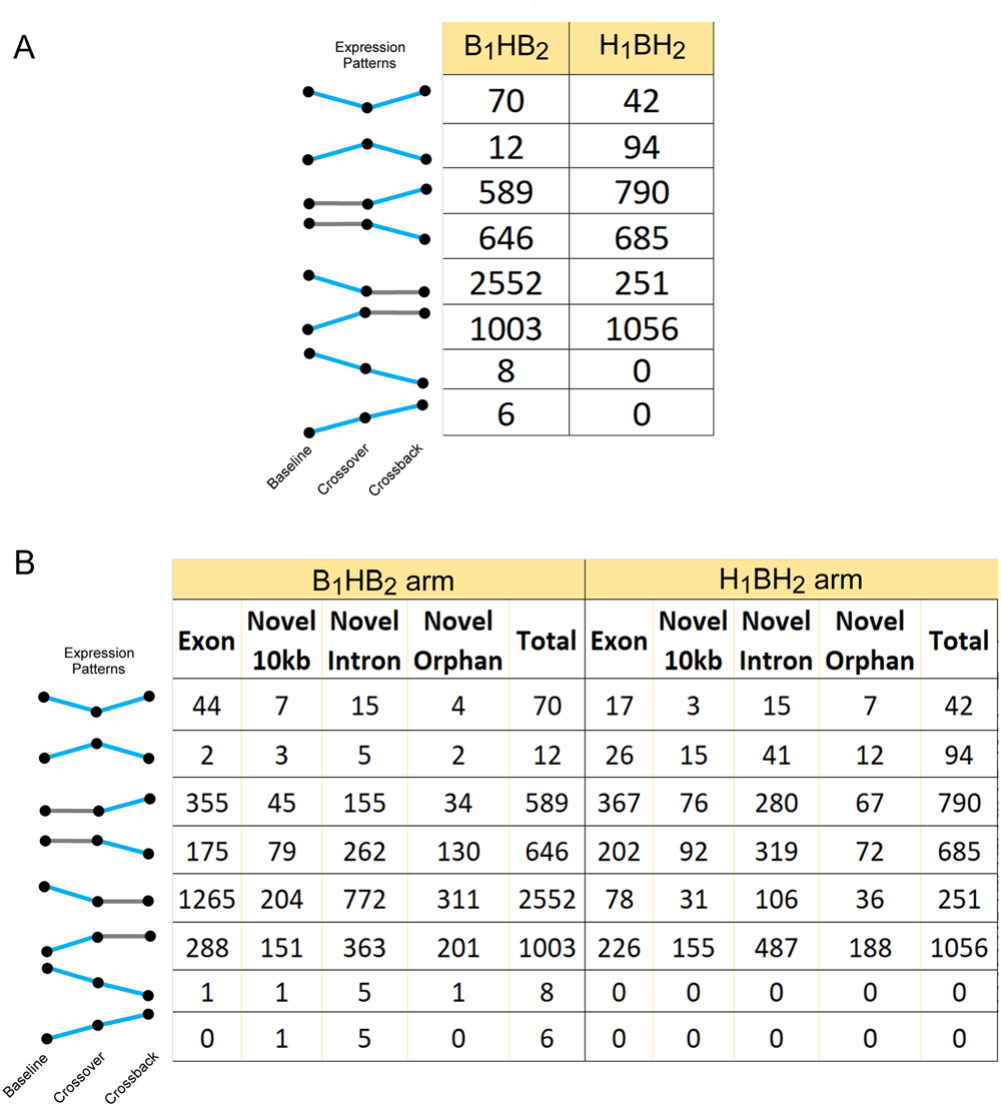
Expression patterns of REs altered across MARS study arms. (**A**) Total RE count for a given expression pattern is shown. Expression patterns are presented to the left of the table, with blue lines indicating significant changes and grey lines indicating non-significant expression changes, measured as a function of slope.

The majority of all REs were exonic (321,207 REs), with intronic REs being the most numerous of the novel classes (Near-exon: 9,730 REs; Intronic: 30,853; Orphan: 10,015). Correspondingly, the majority of REs altered by DBP were either exonic or intronic (Fig. 3). However, novel REs, highlighted by intronic REs, are major components of all observed transcript patterns, indicating that DBP exposure(s) affects far more than known transcripts. Few REs were differentially expressed in both study arms (H_1_BH_2_ arm and B_1_HB_2_ arm), suggesting that the alternating DBP exposures affects DBP-naïve males differently from males chronically exposed to high-DBP mesalamine. This arm-specific effect was also observed in sperm motility and hormonal responses of the MARS individuals^5–7^.

Differential REs were then classified into specific response patterns, defining REs which either changed explicitly with high-DBP exposure. They included acute response REs that changed from baseline to crossover, followed by an opposite recovery response in REs that changed from crossover to crossback, and REs that increased or decreased across all study visits. As summarized in Fig. 3, both study arms had relatively few REs altered with DBP exposure across both transitions (1.7% and 5% in the B_1_HB_2_ arm and H_1_BH_2_ arm, respectively). Unexpectedly, the majority of differential REs in either study arm were significantly altered in a single comparison (i.e., baseline to crossover - acute response or crossover to crossback - Recovery).

Semen analysis of the MARS subjects^5^, showed a continuous decline in sperm motility due to a carry-over effect of high-DBP exposure in the B_1_HB_2_ arm. To examine how sperm motility RNAs may reflect this phenotype, genes known to be involved in sperm motility in mouse were overlapped with the differential exonic REs. Interestingly, several exons of sperm-motility associated genes (ATP1A4, WDR66, TEKT2, TEKT5, DRC7, CFAP44, DDX4, DNAJA1) were downregulated in the B_1_HB_2_ arm, during the initial high-DBP insult (Supplementary Table S5), consistent with the B_1_HB_2_ arm’s observed decline in sperm motility^5^. Several of the aforementioned downregulated genes are known sperm structural components (TEKT2, TEKT5, DRC7, CFAP44)^49–52^. In contrast, in the H_1_BH_2_ arm, semen parameters (including sperm motility) did not change across the study visits and few sperm-motility associated genes were linked with differential exonic REs in the H_1_BH_2_ arm. Notably, a small number of B_1_HB_2_ arm REs that were not directly associated with motility continuously increased (6 REs) or decreased (8 REs) across the study visits, while none of the H_1_BH_2_ arm REs followed a continuous pattern.

### Biological response to DBP exposure

As we have shown in other systems, one would expect spermatozoal RNAs to reflect the biological processes impacted by DBP exposures^53–55^. Towards this end, the genes overlapping the RNAs altered by DBP exposures were assessed initially by Gene Ontology (GO) enrichment. The gene names associated with differential exonic, novel near-exon, or novel Intronic REs in the expression patterns of interest were compiled to resolve GO categories associated with both novel REs and exonic REs. Supplementary Table S6 summarizes the primary affected signaling and literature-based pathways. Few of the top GO pathways were shared between the two study arms, suggesting that shifts in DBP levels differentially affect DBP-naïve males compared to chronically exposed males. Within the B_1_HB_2_ arm, acutely downregulated REs were associated with “RAN-GAP cycling”, “Focal adhesion kinase signaling”, and “Ras GTPase binding”. RAN-GAP cycling, which is a critical component of nucleo-cytoplasmic transport, is likely involved in epigenetic regulation during spermatogenesis, via movement of regulatory RNAs^56^. During mammalian spermiogenesis, Ran GTPase may mediate kinesin localization, which is necessary for spermiation^57,58^. Interestingly, REs upregulated and downregulated in recovery phase of the B_1_HB_2_ arm were enriched for NGF signaling. NGF protein is found throughout male reproductive tissues^59–62^, including mammalian spermatozoa^63^, and outside of the suspected regulatory roles in Sertoli cells^64^, likely facilitates sperm motility^65,66^. A disruption of the NGF signaling-mediated motility in the B_1_HB_2_ arm is congruent with previously noted decreased motility^5^ after administration of a high-DBP mesalamine to DBP-naïve participants.

Within the H_1_BH_2_ arm, acute response REs were associated with organellar organization, which is requisite for extreme cellular and nuclear remodeling during spermatogenesis. Recovery in the H_1_BH_2_ arm suggested an involvement of “Coregulation of androgen receptor”, as well as organelle and chromatin organization. Taken together, the above GO analysis suggested that signaling pathways involved in throughout spermatogenesis, such as NGF signaling, RAN cycling, and androgen receptor signaling, may be altered due to DBP exposures. Concerted activation or repression of signaling pathways was then examined by Ingenuity Pathway Analysis (IPA) to both resolve the enrichment of signaling pathways and identify the relative direction of pathway modulation (Supplementary Fig. S1).

The transition from baseline (H_1_ visit) to crossover (B visit), within the H_1_BH_2_ arm, did not produce any enriched pathways with notable activation or repression. However, the transition from crossover (B visit) to crossback (H_2_ visit), which represents the return to high-DBP mesalamine, activation of NGF signaling (Z = −1.6) was observed, consistent with the previous ontological enrichment analyses. In addition, activation of oxidative stress was indicated through mild activation of ATM signaling (Z = 0.4) and strong activation of nitric oxide production (Z = 2.3). While sumoylation and integrin-linked signaling (ILK), are repressed (Z = −1.1) and activated (Z = 1.7), respectively, both pathways have pleotrophic effects. This is consistent with previous reports of DBP-induced spermatozoal damage and oxidative stress^14,15^. However, the B_1_HB_2_ arm’s enriched pathways do not strongly implicate oxidative stress and a DNA damage response. The transition from baseline (B_1_ visit) to crossover (H visit) is associated with several spermatogenesis-related pathways, including activation of EIF2 signaling (Z = 1.9) and the PPAR-alpha/RXR-alpha signaling (Z = 2.1). These pathways were not strongly associated with either the H_1_BH_2_ arm or in the H visit vs B_2_ visit comparison of the B_1_HB_2_ arm, suggesting that a concerted shift in the PPAR-alpha and EIF2 pathways only occurs upon the initial high-DBP exposure. This is consistent with the current tenant that the detrimental effects of peroxisome proliferators, i.e., DBP, on germ cells likely acts through Sertoli cells^1–3,67^. Currently, the biological implication of repressing NF-KB signaling (Z = 1.9) and 3-phosphoinositide degradation (Z = 2.7) in the context of spermatogenesis is unknown.

In comparison, the transition from crossover (H visit) to crossback (B_2_ visit) revealed a different repertoire of enriched pathways, as well as a strong activation (Z = 2.1) of GP6 signaling^68,69^. Additionally, the retinoic acid receptor (RAR) pathway, which is a known mediator of germ cell differentiation^70^, was also enriched, and although no concerted activity was observed, levels of several of the altered pathway members (CARM1, SWI/SNF, NCOR1, PKC) were consistent with an activation of the retinoic acid nuclear receptor (RAR) and retinoid receptor (RXR) (via binding of retinoic acid). Notably, several of the RAR’s altered pathway members (CARM1, SWI/SNF, NCOR1) associated with changes in chromatin structure^71–76^ have the potential to mediate epigenetic effects of intergenerational inheritance.

### DBP exposure promotes expression of simple repeats

**Figure 4:**
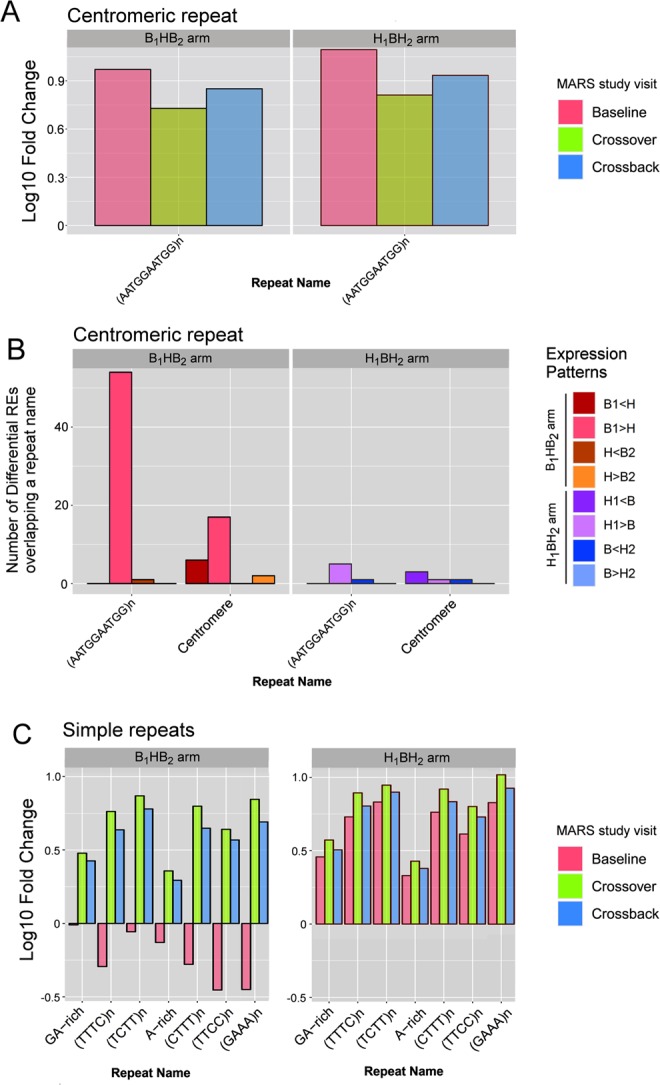
Enrichment of repetitive element expression. (**A**) Repeat enrichment (positive log10 fold change) or depletion (negative log10 fold change) of centromere-associated repeats. X-axis provides the repeat name, while the Y-axis indicates the relative enrichment (positive log10 fold change) or depletion (negative log10 fold change). (**B**) The number of differential REs that overlap centromeric repeats. X-axis provides the repeat name, while the Y-axis indicates the number of differential REs for each significant expression change. (**C**) Repeat enrichment (positive log10 fold change) or depletion (negative log10 fold change) of Simple repeats. High-DBP exposure within the past spermatogenic cycle enriches simple repeats in spermatozoa.

Several classes of repetitive element associated RNAs, including simple repeats, endogenous retroviruses, and centromeric RNAs, have been identified within the population of human spermatozoal RNAs^31,77^. The effect of high-DBP exposure in spermatozoa for each study arm and study visit was assessed as a function of relative enrichment/depletion of REs that overlapped a genomic repeat. Centrometric repeats and MER1A are enriched in mature human spermatozoa (Fig. 4A and Supplementary Fig. S2) in accord with previous studies^31^. As expected, the centromeric repeat, (AATGGAATGG)n, is enriched across all MARS study arms (see Fig. 4A), with no distinct differences across the study arms. This centromeric enrichment is highlighted by novel orphan REs. Interestingly, the abundance of differential REs overlapping the (AATGGAATGG)n repeat decreases from the B_1_ visit to the H visit. As shown in Fig. 4B, this suggests that in the B_1_HB_2_ arm, the transition to high-DBP exposure reduces the levels of (AATGGAATGG)n-associated REs. Centromeric RNA has been shown to facilitate the localization of nucleoproteins and the chromosomal passenger complex (CPC)^78,79^. Interestingly when nuclear structures are resolved, sperm centromeres are located towards the nuclear periphery^80^. This is consistent with the view that centromeric repeat RNA may represent residual transcripts that, in some manner, guide sperm differentiation and/or guide mitotic progression of the early human embryo.

Simple repeats, such as GA-rich repeats and variations of TC-rich repeats (e.g. (TTTC)n) were highly enriched across many of the MARS sperm samples as shown in Fig. 4C. Interestingly, simple repeats were highly enriched in all study visits and arms, with the exception of the B_1_ visit of the B_1_HB_2_ study arm. Within the MARS study set, the B_1_ visit is the sole timepoint for which sperm samples have not been exposed to high-DBP mesalamine. All novel RE classes (near-exon, intronic and orphan), but not the exonic REs, exhibit this DBP-specific pattern of repeat enrichment. Differential repeat analysis verified this DBP-specific pattern for several of the simple repeats (Supplementary Fig. S3), with the primary exceptions of GA-rich and A-rich repeat classes. These results suggest that high-DBP exposure elicits an immediate and acute response, represented by a dramatic increase in the expression of simple repeats in the male gamete. This showed that, in addition to being enriched in spermatozoa, RE-associated genomic repeats are selectively modified by DBP exposure. As these repeats are compartmentalized in sperm^77^, perhaps they also have a role in sperm chromatin organization. In this manner, their modification by DBP, that is known to increase DNA nicking^81^, may specifically alter chromatin states^82^.

### Small RNAs altered by DBP exposure

**Figure 5:**
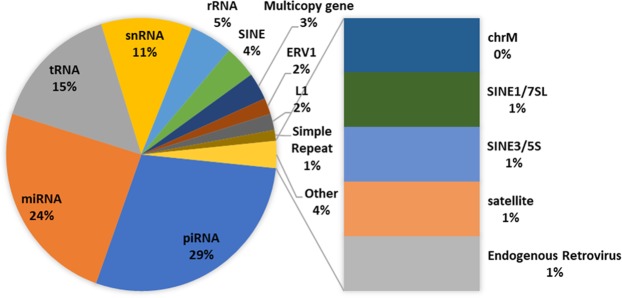
Distribution of small RNA families in highly expressed small RNAs. Highly expressed small RNAs were defined as having a median RPM exceeding 50 across all MARS sperm samples.

Several types of small RNAs, such as miRNAs and piRNAs, have known roles in regulating mRNA and transposable element-derived RNAs and levels of repetitive elements. Accordingly, small size-selected RNA (sncRNA)-Seq libraries, were prepared and sequenced to assess the impact of DBP exposure. Their distribution is summarized in Fig. 5. The 156 highly abundant sncRNAs exceeding a median RPM of 50 are similar to what we and others have established^83–85^, with piRNAs being the most abundant of the sncRNAs (Supplementary Table S7).

**Figure 6:**
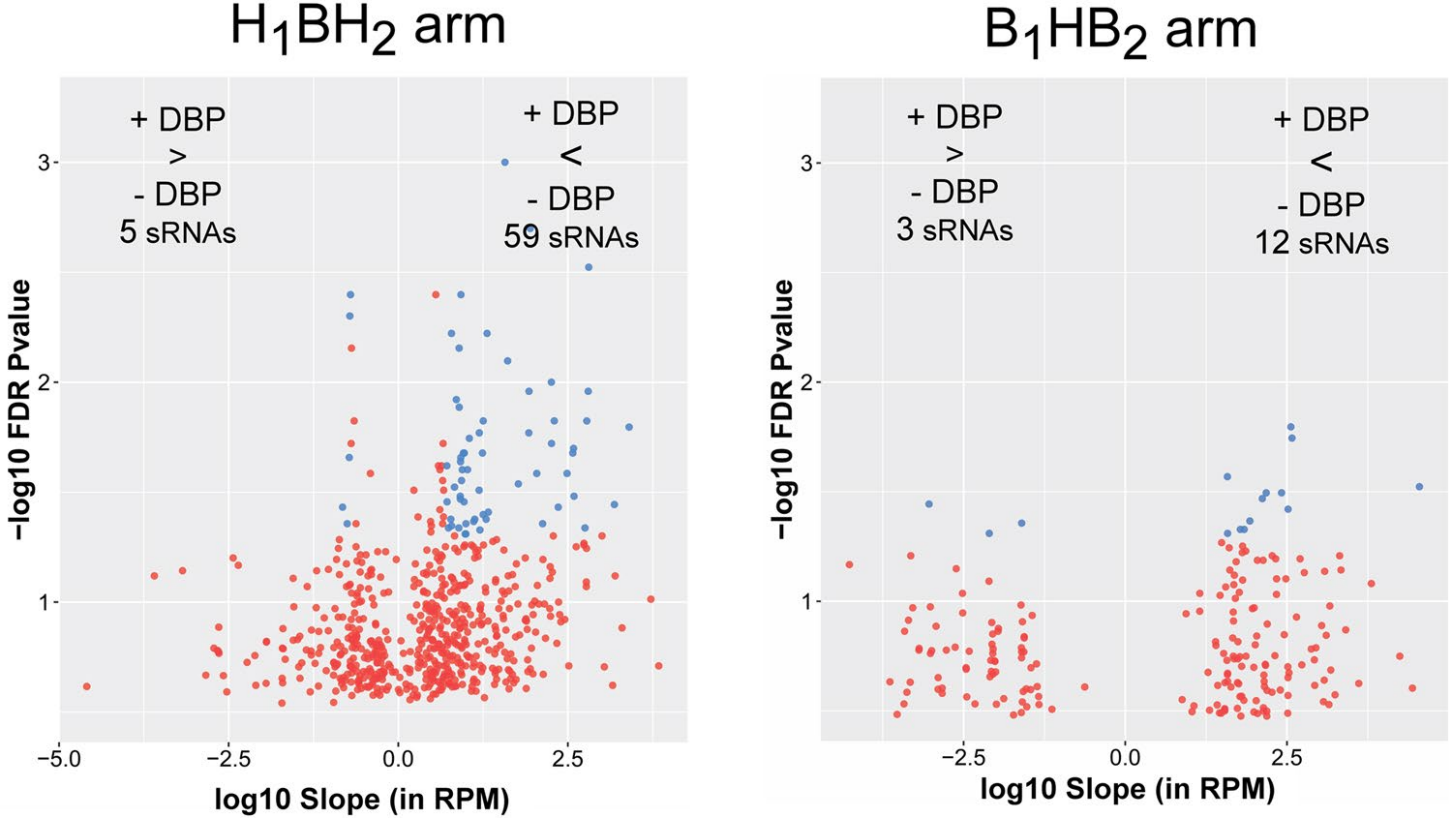
Volcano plots of differential small RNAs. The left and right panels show the volcano plots for the H_1_BH_2_ arm and the B_1_HB_2_ arm, respectively. The X-axis indicates the log10 expression change (slope) in RPM, while the Y-axis indicates the negative log10 empirical P-value.

A LMEM was also applied to the individual study arms to assess the impact of DBP exposure on small spermatozoal RNAs under high-DBP and background-DBP conditions across the entire arm. This approach did not differentiate between study visits (e.g. baseline, crossover, and crossback), but instead treated samples as replicates of the associated high-DBP or background-DBP conditions. This use of this approach was necessary due to small sample sizes of the individual study visits. Both the use of an empirical P-value and Benjamini-Hochberg correction produced a similar number of significant small RNAs. For concordance with adjustment strategy employed in the long RNAs, an empirical P-value was used for the small RNAs. As shown in Fig. 6 and detailed in Supplementary Table S8, in comparison to non-DBP medication, the B_1_HB_2_ arm showed upregulation of 3 small RNAs in response to high-DBP mesalamine and downregulation of 12 small RNAs. In the H_1_BH_2_ arm, exposure to high-DBP mesalamine upregulated 9 small RNAs and downregulated 77 small RNAs. The difference in detection of differential small RNAs between study arms is likely due to the smaller sample size of the B_1_HB_2_ arm. CHARLIE3, a hAT-Charlie DNA transposon, and MER54 were differentially regulated in both study arms. Of note, CHARLIE3 was upregulated in the B_1_HB_2_ arm, yet down regulated in the H_1_BH_2_ arm upon high-DBP exposure. In contrast, MER54 was down regulated in both study arms upon high-DBP exposure. Endogenous retroviruses of the ERV3 class, of which MER54 is a member, have been previously observed in human reproductive and embryonic tissues, including placenta^86,87^. Currently, the phenotypic impact of a DBP-induced reduction in ERV3 RNAs during spermatogenesis and embryogenesis is not known.

## Discussion

In both rural and urban environments, humans are exposed to cocktails of endocrine disruptors^88^. While environmental regulations designate maximum allowable levels of only a subset of the numerous xenobiotics, this level is primarily determined through animal models (including rat and mouse), which may not accurately reflect the human condition^89^. The human male is known to mediate some intergenerational effects in offspring^19^, yet the intergenerational effect of adult paternal exposures to common xenobiotics and endocrine disruptors, particularly in humans, is poorly characterized. The MARS study showed that exposure of human males to high levels of a single endocrine disruptor, di-butyl phthalate (DBP), is capable of reducing sperm motility in DBP-naïve subjects^5^.

Applying RNA-seq to the MARS analysis (Fig. 1) showed that DBP-induced alterations in spermatozoal RNAs were largely unique to a single study arm (either the acute B_1_HB_2_ or chronic H_1_BH_2_ study arm). Each biological response to increasing or decreasing DBP levels yielded a different altered RNA profile (Fig. 3). Interestingly, novel RE’s comprise a significant portion of altered REs, indicating that DBP exposure(s) affects far more than the previously known transcripts. The RNA profiles observed in the spermatozoa ejaculate reflect the final outcome of spermatogenesis, which includes both RNAs generated in preparation for differentiation and those acquired during epididymal maturation, also for transmission to the future embryo. Within the immunoprivileged testis (reviewed in^90^), the spermatogenic effect of high-DBP mesalamine is likely communicated through Sertoli cells that support the germline during differentiation, or through the epididymis during transit, when the sperm first become exposed to other fluids.

Cell lines have previously indicated that phthalate exposure may be acting on reproductive tissues through processes that include PPAR-dependent mechanisms^1–3^, inducing oxidative damage^14,15^. In the current study, several spermatogenesis-related pathways, including activation of EIF2 signaling and the PPAR-alpha/RXR-alpha signaling, were strongly associated with the transition from baseline (B_1_ visit) to crossover (H visit) (Supplementary Fig. S1). This suggests that a concerted shift in the Peroxisome Proliferator-Activated Receptor alpha (PPAR-alpha) and Eukaryotic Initiation Factor 2 (EIF2) pathways occurs at the initial high-DBP exposure in DBP naïve males. Conversely, within the H_1_BH_2_ arm, after a temporary respite of a single spermatogenic cycle on non-DBP medication, the return to high-DBP mesalamine activated oxidative stress and DNA damage response pathways, perhaps signaling the beginning of the repair process (Supplementary Fig. S1). The ontological association of androgen receptor coregulation in the H_1_BH_2_ arm of the MARS study also suggests that responses to *in vivo* adult exposures elicits androgen disruption, which has previously been implicated in phthalate-induced testicular dysgenesis syndrome. Notably, subjects in the H_1_BH_2_ arm have been chronically exposed to high-DBP mesalamine, some for several years, prior to the MARS study and are assumed to have reached a phthalate-induced expression plateau in response to the high-DBP levels. The temporary (1 spermatogenic cycle) withdrawal from high-DBP mesalamine then precedes the additional and significant stress imparted onto the germline upon re-introduction of high-DBP mesalamine. The initial changes that DBP triggers are currently undefined, as ejaculated spermatozoa serve in this study as a proxy for testicular function. However, the initial prompt to change is likely an effect of DBP on Sertoli cells and/or the supporting testicular cell types, such as Leydig cells. In both study arms, the results suggested that the processes by which chronic high-DBP exposure modifies testicular function are different from those processes needed to recover from the exposure.

Among the genomic repetitive elements shown to be enriched in spermatozoa, TC-rich tetramers form a larger contribution to the sperm RNA when an individual has experienced a high-DBP exposure in the previous spermatogenic cycle (Fig. 4). The time required to fully recover is not known and may well be far longer than the single crossback cycle observed in the B_1_HB_2_ arm of the current study. Nevertheless, the study indicates that any recent high-DBP exposure increases the abundance of simple repeats in human spermatozoa. The biological function of these recurring simple repeats in spermatogenesis, fertilization and early embryo development has yet to be categorically defined^31^. However, they are compartmentalized in sperm^77^, perhaps reflective of a role in sperm chromatin organization. Phthalate’s noted ability to increase DNA nicking^81^ and thus spermatozoal DNA fragmentation^14,15^ in a specific manner^82^ may also alter the specialized compact chromatin environment in spermatozoa. Currently, the physiological effect(s) of the cocktail of background exposure to endocrine disruptors in humans, particularly in somatic tissue, is unknown. However, given the potential intergenerational effects of the paternal germline (mediated by sperm RNA content and sperm epigenome), ubiquitous human exposure to phthalates and other known endocrine disruptors remains a concern.

The MARS study also provided the opportunity to assess the spermatozoal impact of mild Inflammatory Bowel Disease (IBD). IBD, defined as Ulcerative colitis and Crohn’s disease, is a common condition, with a prevalence of approximately 1 per 500 people^91^. The current study compared males with non-flaring, mild IBD treated daily with mesalamine to a control cohort of fertile males from idiopathic infertile couples. As expected, mild IBD, or chronic mesalamine use, had minimal impact on spermatozoal RNAs (Fig. 2).

At present, the MARS study demonstrates that exposure of human males to high levels of a single endocrine disruptor, di-butyl phthalate (DBP), is capable of altering spermatozoal RNAs and expression of genomic repeats in sperm. Furthermore, an individual’s history of high-DBP exposure influences their reproductive response to changes in DBP levels. The time period required to fully recover from a high-DBP exposure, if it is possible, has yet to be determined. However, this study suggests that recovery is at least longer than a single spermatogenic cycle (approximately 90 days). Future *in vitro* and *in vivo* experiments relevant to adult phthalate exposures are required to identify the mechanisms and pinpoint the biological processes at work in the reproductive and endocrine systems of phthalate-exposed adults. Observational studies of offspring from DBP-exposed fathers will provide a path to determine the extent of the impact that paternal DBP exposure presents as health risk to their subsequent children.

## Materials and Methods

The use of human tissues was approved by the Wayne State University Human Investigation Committee and carried out under Wayne State University Human Investigation Committee IRB Protocol 095701MP2E(5R) in accordance with relevant guidelines and regulations after informed consent was obtained from the collection sites. The study was approved by the institutional review boards Partners (MGH) protocol number is 2005P001631 of Harvard T.H. Chan School of Public Health, BIMC, BWH and MGH. All men signed informed consents.

### Sperm purification and RNA-seq library construction

RNA isolation and RNA-seq library construction was as essentially described^34,92,93^. In brief, to construct long RNA-seq libraries, after RNA isolation and DNase treatment, two nanograms of RNA per sample was subject to Seqplex (Sigma-Aldrich) amplification. Fifty nanograms of Seqplex cDNA product was then subjected to NEBNext Ultra DNA Library construction (New England Biolabs) to create sequencing libraries. All samples were subject to paired-end sequencing using either the NextSeq 500 (Illumina) sequencer, HiSeq 2500 (Illumina) sequencer or the HiSeq 4000 (Illumina) sequencer.

To construct small RNA-seq libraries, one nanogram of small RNA per sample was subjected to the NEXTflex Small RNA-Seq v2 (Bioo Scientific) protocol as detailed by the manufacturer. Complete barcoded libraries were quantitated and pooled for sequencing. Samples were subject to paired-end sequencing using the MiSeq (Illumina) sequencer.

### RNA-seq data processing methods

Sperm RNA-seq datasets used as the control cohort from couples who had a Live Birth were downloaded from the Gene Expression Omnibus (GEO), accession number GSE65683^34^. MARS long RNA libraries were processed similarly to the GSE65683 samples. Paired-end reads were trimmed of adaptors and low-quality bases prior to alignment to the consensus human ribosomal RNA (GenBank: U13369.1), the human genome (hg38) and exogenous RNA spike-in sequences, using HISAT2 (version 2.0.6). Read alignments to the human genome, exogenous RNAs, and U13369.1 were processed to remove duplicated reads using Picardtools MarkDuplicates (version 1.129). Poorly performing MARS samples were removed after examination of alignment statistics and similarity to human testicular RNA-seq from GTEx (https://www.gtexportal.org/home/) and sperm RNA veracity was assessed as described (Supplementary Materials) allowing for the quantitative classification of samples.

RNA element (RE) discovery algorithm, REDa, (described in^31^) was applied to the MARS and GSE65683 (control) samples. Expression (in Reads Per Kilobase per Million - RPKM) for the RE loci was then calculated for all MARS and GSE65683 samples.

### Small RNA

MARS small RNA libraries were trimmed of adaptors and low-quality bases, followed by removal of reads smaller than 13 bp. sncRNAbench (version 10.14)^94^ was used for assigning reads to small RNA types and repeat classes, followed by a custom code for generating normalized expression values (RPM- Reads Per Million). Based on a yield of 0.3 fg of sncRNA/sperm cell, a threshold of 50,000 input reads for subsequent analysis were required of which 94% of the samples fulfilled. Common types of small RNAs of interest, such as miRNAs, piRNAs, tRNAs, tRNA fragments, and siRNA can be detected with small RNA libraries (miRNAs ~22 bp)^95^, piRNA ~24–31 bp^96^, and tRNA fragments ~28- to 34-nt^20^). All small and long RNA libraries are archived (GSE129216).

### Differential long RNAs

The control cohort was composed of 52 subjects from idiopathic infertile couples who sought reproductive care and presented with a live birth^34^. The control samples are assumed to be free of inflammatory bowel disease or ulcerative colitis, and are thus considered “Normal” in differential analyses. To identify REs modified by IBD, a linear model was used to compare the Normal sperm to the B_1_HB_2_ arm of the MARS study, with three total comparisons being performed (Normal vs B_1_; Normal vs H; Normal vs B_2_), each accounting for influential covariates and subject characteristics. Benjamini-Hochberg multiple-testing correction was applied. Significance was assigned to an RE if the absolute value of the slope exceeded 10 RPKM and the Benjamini-Hochberg adjusted P-value was less than 0.05. REs modified in a consistent manner (e.g. IBD-enriched or control-enriched) in any two of the three visits (B_1_, H, and B_2_) were considered for further investigation.

To identify REs modified by DBP exposure, a Linear Mixed-Effects Model (LMEM) was used to detect REs that changed with DBP exposure. Models were applied to each study arm independently, accounting for influential covariates and subject characteristics. Two comparisons were carried out for each study arm, in order to identify changes occurring from baseline visit to crossover visits, and again from crossover visit to crossback visit. Due to the large number of REs (>100,000) tested in each comparison, an empirical (bootstrapped) P-value was generated using random resampling.

REs were subsequently classified into eight unique expression patterns, with significance determined if the absolute value of the slope exceeded 10 RPKM and the empirical P-value was less than 0.05. Differential REs were then classified into specific response patterns, defining REs which either changed explicitly with DBP exposure, i.e., acute response REs that changed from baseline to crossover visits, followed by an opposite recovery response in REs that changed from crossover to crossback visits (see top two panels of Fig. 3A). Patterns of acute change were defined as those where a RE was significantly up- or down-regulated in the baseline to crossover visit comparison, but not altered from crossover to crossback visit comparison. Patterns of recovery are defined as those where a RE did not change in the baseline to crossover visit comparison, but is significantly up- or down-regulated from crossover to crossback visits. Patterns of additional interest were those that continuously increased across the study arms or continuously decreased across the study arms.

### Differential small RNAs

Human small RNA sperm samples (<50 bp) used in the study are summarized in Supplementary Table S1, with 85 small RNA libraries used in modeling. A LMEM was applied to identify differential expression between DBP state (high-DBP vs non-DBP mesalamine) for the individual study arms, while accounting for influential covariates and subject characteristics. Multiple testing correction was applied using an empirical (bootstrapped) P-value, generated using random resampling. Both the use of an empirical P-value and Benjamini-Hochberg correction produced similar results of significant small RNAs. For concordance with adjustment strategy employed in the long RNAs, an empirical P-value was used. Differential small RNAs were defined as those whose absolute value of the slope exceeded 5 RPM and empirical P-value was less than 0.05.

### Repeat enrichment

Repeat enrichment was measured using the following formula, where “R” indicates the REs associated with the repeat of interest, “A” indicates the REs associated with any repeat, and the required median expression threshold for a study visit is 25 RPKM. Repeat enrichment is the change in contribution of the given repeat to the repeat population, when a given expression threshold is applied to both the repeat of interest and the total repeat population.

$${\rm{\Delta }}\,ratio=\,\frac{\#\,{R}_{expressed}}{\#\,{A}_{expressed}}-\frac{\#\,R}{\#\,A}$$

The statistical significance of an enrichment or depletion (indicated with a positive or negative ∆ ratio, respectively) was tested using a hypergeometric test, implemented in the *stats* R package. This method is similar to that implemented in Estill *et al*.^31^. The repeat enrichment analysis merely indicates if a repeat type is relatively under- or over-represented in the expressed REs, relative to the expected proportion when no expression threshold is applied.

### Ontological enrichment and pathway analysis

Gene Ontology (GO) enrichment was generated using the GeneRanker function of Genomatix (Eldorado version 12-2017), from the Genomatix software suite (https://www.genomatix.de/), version 3.10. The gene names associated with differential exonic, novel near-exon, or novel Intronic REs were compiled and used as input to GeneRanker. Pathway enrichment was assessed using Ingenuity Pathway Analysis (IPA) (version 1–13, Content build 46901286). The expression changes occurring in differential exonic REs were compiled and used as input for IPA. The expression changes of all differential exonic REs belonging to a given gene were averaged. This produced a single slope, p-value, and computed log2ratio for each gene name.

To identify DBP-altered exonic REs originating from genes involved in sperm motility, all murine genes in MGI database with the associated Gene Ontology term “sperm motility” (GO:0097722, http://www.informatics.jax.org/go/term/GO:0097722) were downloaded and transformed into the HGNC (human) gene symbol using custom R code and the BiomaRt package. The gene symbols associated with the differential exonic REs, partitioned according to expression change, were then overlapped with the list of HGNC gene symbols.

## Supplementary information

Supplementary information

## Data Availability

The RNA-seq datasets from this study have been deposited as GEO Datasets with the following accession number: GSE129216.
