# Supplementary material for: The effects of di-butyl phthalate exposure from medications on human sperm RNA among men
Source: Sci Rep. 2019 Aug 27;9:12397. doi: 10.1038/s41598-019-48441-5 (PMC6711971; doi:10.1038/s41598-019-48441-5)
Supplement: Supplementary file 1 — Supplementary information [file 41598_2019_48441_MOESM1_ESM.docx]

**Supplementary Materials**

**Title:** The effects of di-butyl phthalate exposure from medications on human sperm RNA among men

**Authors:** Molly Estill^1^, Russ Hauser^3^, Feiby L. Nassan^4^, Alan Moss^5^, and Stephen A. Krawetz^1,2^

*^1^Center for Molecular Medicine and Genetics, Wayne State University School of Medicine, Detroit, MI 48201, USA;  ^2^Department of Obstetrics and Gynecology, Wayne State University School of Medicine, Detroit, MI 48201, USA;  ^3^Vincent Memorial Obstetrics and Gynecology Service, Massachusetts General Hospital, Harvard Medical School, and Departments of Environmental Health and Epidemiology, Harvard T.H. Chan School of Public Health, Boston, MA, 02115, USA; ^4^ Departments of Environmental Health and Nutrition, Harvard T. H. Chan School of Public Health, MA 02115, USA;  ^5^Department of Gastroenterology, Beth Israel Deaconess Medical Center, Boston, MA, USA*

**Supplementary Methods**

*RNA-seq data processing methods*

A total of 72 RNA-seq datasets were downloaded from the Gene Expression Omnibus (GEO), accession number GSE65683 (*35*). Paired-end reads were trimmed of adaptors and low-quality bases using Trimmomatic (version 0.36) (*98*), using default parameters (2:30:10 LEADING:3 TRAILING:3 SLIDINGWINDOW:4:15), and requiring a minimum read length of 50 bp (MINLEN:50). The TruSeq Universal adaptor (AATGATACGGCGACCACCGAGATCTACACTCTTTCCCTACACGACGCTCTTCCGATCT) was used as input to Trimmomatic. Paired and unpaired reads were aligned to the consensus human ribosomal RNA (GenBank: U13369.1) using HISAT2 (version 2.0.6) and the non-default parameters (-p10 --max-seeds 30 -k 2), then aligned to the human genome (hg38) and exogenous RNA spike-in sequences, using the same HISAT2 parameters. Reads without alignments in either U13369.1, the human genome (hg38), or an exogenous RNA were further assessed for alignments to repeat sequences (RepBase, February 2017 release), using HISAT2, with the following parameters (-p10 --no-spliced-alignment --max-seeds 10 -k 3). Reads without alignments in either U13369.1, hg38, an exogenous RNA, or RepBase were aligned to bacterial and viral genomes using Kraken (version 0.10.5-beta) and Jellyfish (version 1.1.10), implementing the full Kraken library and filtering alignments with a threshold of 0.15. Read alignments to the human genome, exogenous RNAs, and U13369.1 were processed to remove duplicated reads using Picardtools MarkDuplicates (version 1.129).

MARS long RNA libraries were processed similarly to the GSE65683 samples, with necessary modifications to the minimum read length (25 bp) and adaptor sequence (GATCGGAAGAGCACACGTCTGAACTCCAGTCdUACACTCTTTCCCTACACGACGCTCTTCCGATC) during adaptor trimming with Trimmomatic. QC of the MARS study’s long RNA samples was accomplished by examination of alignment statistics, allowing for the quantitative classification of samples failing QC into one of five categories (Category 1: Low genomic alignment, Category 2: High intergenic reads, Category 3: High bacterial and viral reads, Category 4: High spike-in reads; Category 5: High unmapped reads). A custom R code was used for assigning the quality control classification

RNA element (RE) discovery algorithm, REDa, (described in (*32*)) was applied to the MARS and GSE65683 (control) samples. Expression (in Reads Per Kilobase per Million - RPKM) for the RE loci was then calculated for all MARS and GSE65683 samples. Due to the use of REs, rather than whole transcripts, paired-end read alignments were treated as individual (single) reads, and the common FPKM value was replace by RPKM. Depending on a read pair’s insert size and RE length, this approach has the risk of inflating the relative read count for a given RE. To mitigate this risk, read counts were assessed for the forward and reverse reads separately, with the read count from single (non-paired due to mate loss during quality control) reads were added to the forward and reverse read counts. The averaged read count between the forward and reverse reads were then used for generating the RPKM values.

*Differential long RNAs*

When comparing IBD samples to normal samples, the control cohort was composed of subjects from idiopathic infertile couples. To reduce the potential effect of idiopathic infertility on the overall RNA profiles and concurrently maximize the size of the control cohort, only control sperm samples which presented with all live birth (LB) (52 samples) were considered for use in differential expression (*35*). With respect to inflammatory bowel disease or ulcerative colitis, the control samples were then assumed to be disease free, and are thus labeled “Normal” in differential analyses.

To identify REs modified by IBD, a Linear Model (LM) was used to compare the Normal sperm to the B_1_HB_2_ arm of the MARS study, with three total comparisons being performed (Normal vs B_1_; Normal vs H; Normal vs B_2_). The following formula was used for all three comparisons: “lm(value ~ seqset + lib + age + protamine_ct + sigma_ct + RNA_conc + cellcount_millions,data=input_data). Multiple-testing correction was applied as Benjamin-Hochberg. REs modified in a consistent manner (e.g. IBD-enriched or control-enriched) in any two of the three visits (B_1_, H, and B_2_) were considered for further investigation. In the current study, the control samples were all present in a single sequencing batch, so a batch effect would be indistinguishable from the control state. Several of the differential REs initially identified as IBD-enriched were differential solely due to near-zero values in all control samples, suggesting a batch effect, and were subsequently removed from consideration if the mean control expression was less than 1 RPKM. This step removed 6 of the 32 REs enriched in IBD for at least two visits.

To identify REs modified by DBP exposure, a Linear Mixed-Effects Model (LMEM) was used to detect REs that changed with DBP exposure. Models were applied to each study arm independently. Two comparisons were carried out for each study arm, in order to identify the changes occurring from baseline visit to crossover visits, and again from crossover visit to crossback visit. The following formula was applied to the H_1_BH_2_ arm: “rpkm ~ visit_simp + lib + period_asacol + bmi + season_warm + smokstat + age_bq + sigma_ct + percent_genomic_duplicated + percent_genomic + (1 | patient)”. The following formula was applied to the B_1_HB_2_ arm of the MARS study: “rpkm ~ visit_simp + lib + bmi + season_warm + smokstat + age_bq + sigma_ct + percent_genomic_duplicated + percent_genomic + (1 | patient)”. Due to the large number of REs (>100,000 REs) tested in each comparison, standard multiple testing corrections statistical significance could not be resolved. Therefore, a bootstrapped P-value was generated using random resampling. A total of 1,000 iterations were performed by permuting the RE expression value and running the given model, while maintaining the same sample order and covariate order. The empirical P-value was thus defined as the proportion of the 1,000 random iterations that resulted in a P-value less than the original P-value.

$Empirical P.value=1- \frac{{\# iterations}_{P.value>P_{0}}}{\# iterations}$

REs were subsequently classified into eight unique expression patterns, with significance determined if the absolute value of the slope exceeded 10 RPKM and the empirical P-value was less than 0.05. REs that changed in tandem with DBP exposure would display a pattern of increased expression from baseline to crossover visits, then decreased expression from crossover to crossback visits, or vice versa (see top two panels of **Table 6A**). Patterns of acute change were defined as those where a RE was significantly up- or down-regulated in the baseline to crossover comparison, but not altered from crossover to crossback. Patterns of recovery were defined as those where a RE is unchanged in the baseline to crossover comparison, but is significantly up- or down-regulated from crossover to crossback. Patterns of additional interest were those that continuously increased across the study arms or continuously decreased across the study arms.

*Differential small RNAs*

The human sperm samples utilized for small RNAs (<50 bp) are enumerated in **Supplementary Table S1**. A total of 81 small RNA sample libraries were subsequently used in modeling, using a LMEM. Due to the low power in the B_1_HB_2_ study arm and insufficient numbers of complete trios, the predictive variable used was the DBP state (e.g. high- DBP or non-DBP mesalamine), whereas the long RNA analysis used the study visit as the predictive variable. The formula used for both study arms’ small RNAs was “rpm ~ med_simp + bmi + season_warm + age_bq + sigma_ct + percent_genomic_duplicated + percent_genomic + (1 | patient)”. In this formula, the influence of medication (high or background DBP) on small RNA expression was corrected for patient BMI, seasonal warmth, patient age, sigma_ct from long RNA libraries, genomic duplication rate from the long RNA libraries, and proportion of long RNA reads aligning to the autosomal and sex chromosomes. Multiple testing correction was applied using a bootstrapped P-value, generated using random resampling. 1000 iterations were performed by permuting the RE expression value and running the given model, while maintaining the same sample order and covariate order. The empirical P-value was thus defined as the proportion of the 1000 random iterations that resulted in a P-value less than the original P-value. Both the use of an empirical P-value and Benjamini-Hochberg correction produced similar results of significant small RNAs. For concordance with the multiple testing adjustment strategy employed in the long RNAs, an empirical P-value was used. Differential small RNAs were defined as those whose absolute value of the slope exceeded 5 RPM and empirical P-value was less than 0.05.

**Supplementary Figures**


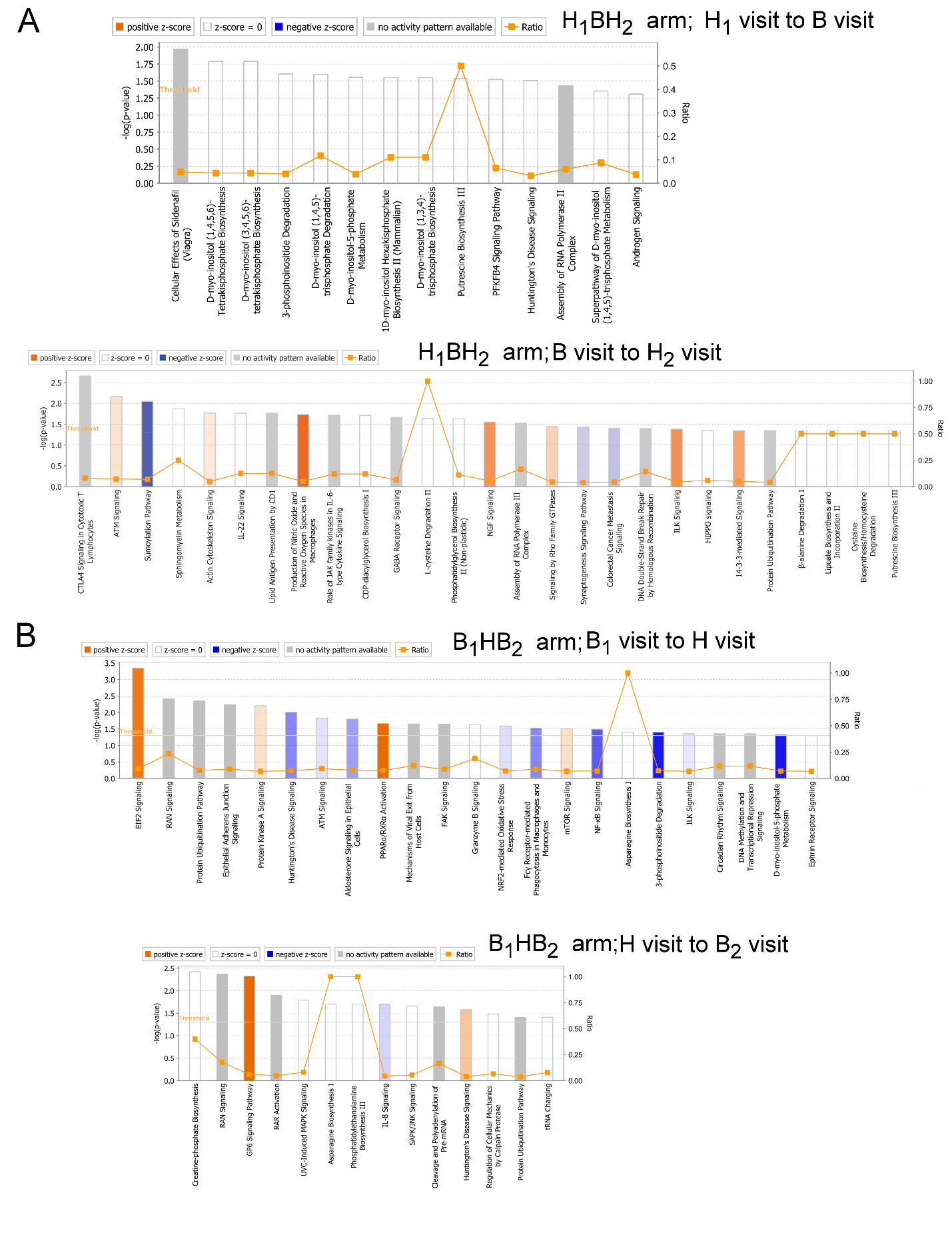


**Fig. S1. IPA pathways of REs altered across MARS study arms.** (A) Pathway enrichment for H_1_BH_2_ study comparisons. (B) Pathway enrichment for B_1_HB_2_ study comparisons. Enriched pathways are ordered according to the relative significance, with most significantly enriched pathways displayed on the left. Pathways highlighted in orange hues and blue hues indicate predicted pathway activation or repression, respectively, with darker colors indicating greater confidence in the activation/repression prediction. Briefly, pathway activation/repression prediction is based on the correlation of inputted gene expression changes with the pathway’s known activity patterns.

**
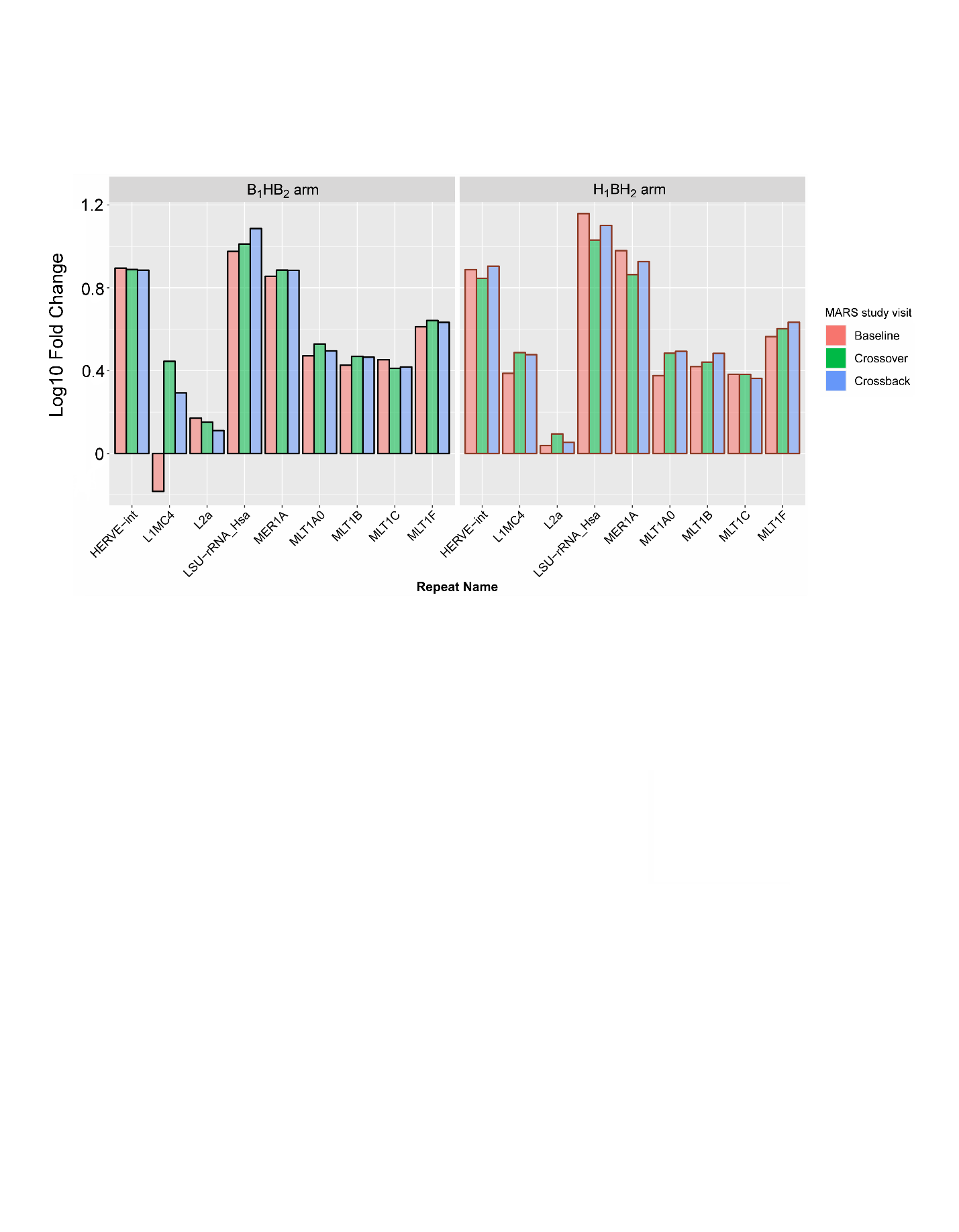
**

**Fig. S2. Enrichment of complex repeats.** X-axis provides the repeat name, while the Y-axis indicates the relative enrichment (positive log10 fold change) or depletion (negative log10 fold change). Baseline, crossback and crossover study visits are colored as light red, light blue, and green, respectively.


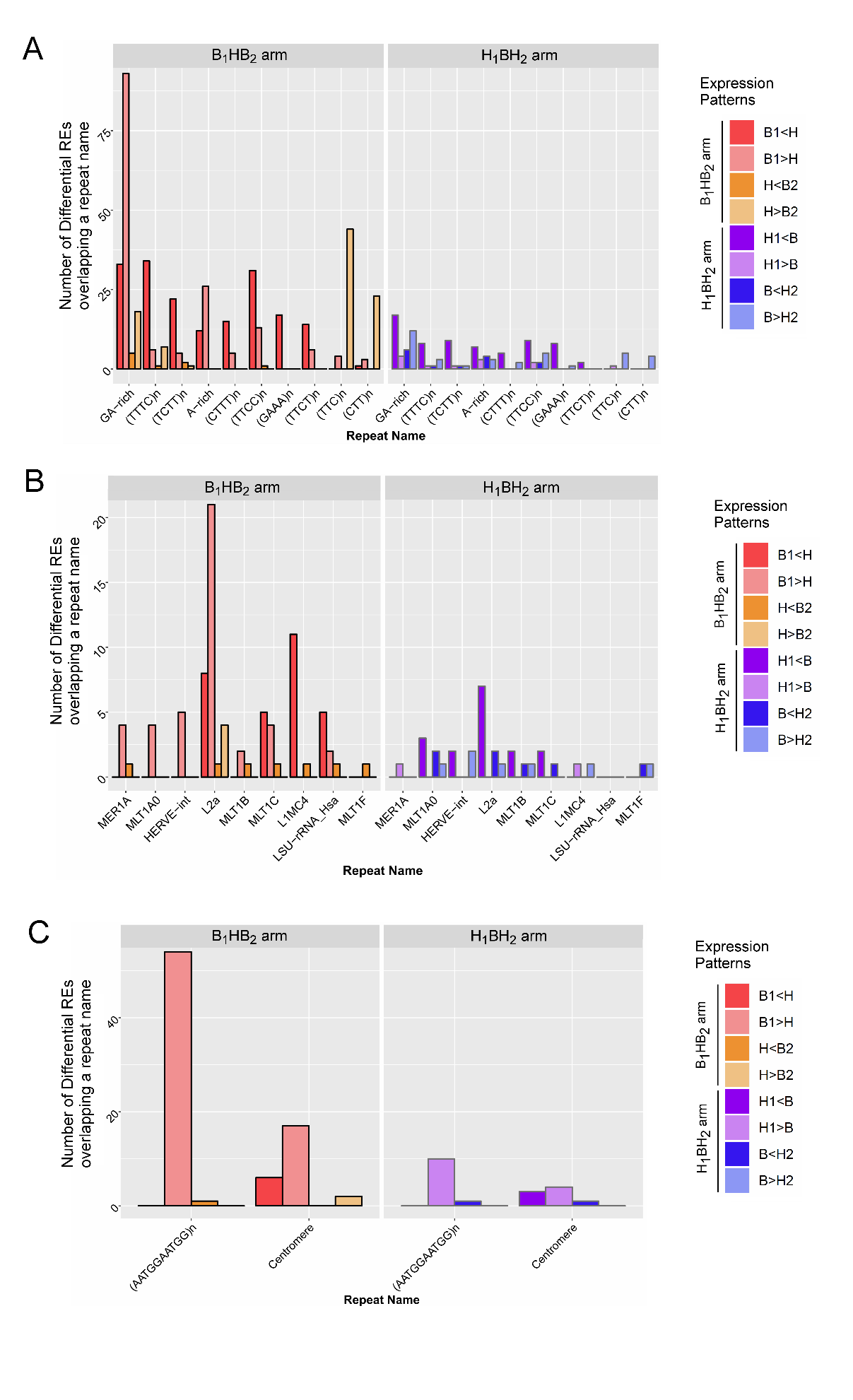


**Fig. S3. Differential expression of sperm-enriched genomic repeats.** The number of differential REs that overlap a given repeat are indicated for (A) Simple repeats, (B) Complex repeats, and (C) Centromeric repeats. X-axis provides the repeat name, while the Y-axis indicates the number of differential REs for each significant expression change.

**Supplementary Tables**

**Table S1. Summary of sample quality.** The first two columns (“Study arm” and “RNA type”) indicate the study arm(s), and RNA type (small RNA or Long RNA library) for the given table row. “Total Samples” and “Pass QC samples” indicate the number of RNA-seq samples sequenced and the number of sequenced samples that passed quality control, respectively. “Subjects with samples” and “Subjects with pass QC samples” indicate the number of patients for which sequenced samples and sequence samples that passed quality control measures, respectively, were available. “Average pass QC samples per person” indicates the average number of sequence samples that passed quality control measures per patient. “Subjects with a Pair/trio” indicate the number of patients for whom the sequenced samples that passed quality control measures formed either a continouous pair (baseline & crossover visits or crossover & crossback visits) or a visit trio (baseline, crossover, and crossback).

| Study arm | RNA type | Targeted Insert Size | Total Samples | | Pass QC samples | Subjects with samples | Subjects with pass QC samples | Average pass QC samples per person | Subjects with a Pair/trio |
| --- | --- | --- | --- | --- | --- | --- | --- | --- | --- |
| H_1_BH_2_ arm | Long RNAs | 150 bp | | 124 | 91 | 27 | 26 | 3.5 | 19 |
| B_1_HB_2_ arm | Long RNAs | 150 bp | | 82 | 59 | 21 | 21 | 2.8 | 16 |
| All arms | Long RNAs | 150 bp | | 206 | 150 | 48 | 47 | 3.5 | 35 |
| H_1_BH_2_ arm | Small RNAs | 13-50 bp | | 62 | 57 | 21 | 21 | 2.7 | 11 |
| B_1_HB_2_ arm | Small RNAs | 13-50 bp | | 24 | 23 | 12 | 12 | 1.9 | 6 |
| All arms | Small RNAs | 13-50 bp | | 84 | 80 | 33 | 33 | 2.4 | 17 |

**Table S2. Highly expressed REs across the MARS study.** The top 50 most expressed REs, ordered from highest RPKM to lowest, are listed. For REs that encompass more than one transcript, the gene names are separated by a comma. REs highlighted in bright yellow indicate a series of genes that are known to be primarily expressed in testis and sperm.

| Element Identifier | Gene Symbol | Median expression across  MARS study | Note |
| --- | --- | --- | --- |
| chrM_577_3304 | MT-TF | 352637.5 | Mitochondrial |
| chr6_52995620_52995950 | RF00100,RN7SK | 6053.6 |  |
| chr16_11280836_11281035 | PRM1 | 5693.2 | Protamine 1, Exon 1 |
| chrM_5904_7445 | MT-CO1 | 5124.1 | Mitochondrial |
| chr14_49586579_49586878 | AL139099.4,RN7SL1 | 4284.3 |  |
| chr16_11275639_11275981 | PRM2 | 4272.6 | Protamine 2, Exon 1 |
| chr14_49862550_49862849 | RN7SL2 | 4121.2 |  |
| chrM_7518_8269 | MT-TD | 3233.5 | Mitochondrial |
| chr5_7304232_7304261 | AC091951.1 | 3147.1 |  |
| chr16_11281127_11281350 | PRM1 | 3002.6 | Protamine 1, Exon 2 |
| chrM_8366_14148 | MT-ATP8,MT-ATP6,MT-CO3 | 2886.1 | Mitochondrial |
| chrM_3307_4331 | MT-ND1 | 2672.1 | Mitochondrial |
| chrM_4402_5579 | MT-TM | 2381.4 | Mitochondrial |
| chrM_14149_14742 | MT-ND6 | 1986.0 | Mitochondrial |
| chr1_152878317_152878446 | SMCP | 1905.9 | Sperm Mitochondria Associated Cysteine Rich Protein, Exon 1 |
| chr16_11276100_11276480 | PRM2 | 1887.2 | Protamine 2, Exon 2 |
| chr1_16740516_16740679 | RNU1-4 | 1777.4 |  |
| chr2_216859896_216860064 | TNP1 | 1764.3 | Transition Protein 1, Exon 2 |
| chr9_9442060_9442380 | RN7SL5P | 1737.4 |  |
| chr2_216859458_216859695 | TNP1 | 1601.2 | Transition Protein 1, Exon 1 |
| chrM_14747_15953 | MT-CYB | 1536.0 | Mitochondrial |
| chr12_112267077_112267394 | RN7SKP71 | 1458.7 |  |
| chr5_7303912_7303962 | AC091951.1 | 1425.8 |  |
| chr5_7302224_7302282 | AC091951.1 | 1372.1 |  |
| chr3_15738515_15738809 | RN7SL4P | 1368.6 |  |
| chr6_49712501_49712604 | CRISP2 | 1326.8 | Cysteine Rich Secretory Protein 2, Exon 2 |
| chr2_88788318_88788346 | ANKRD36BP2 | 1279.4 |  |
| chr15_22771692_22771750 | AC011767.1 | 1270.8 |  |
| chr1_16514122_16514285 | RNU1-1 | 1241.7 |  |
| chr14_66488987_66489037 | CCDC196 | 1199.3 |  |
| chr6_49697860_49697957 | CRISP2 | 1193.9 | Cysteine Rich Secretory Protein 2, Exon 8 |
| chr6_34697159_34697470 | AL451165.2 | 1122.3 |  |
| chr14_49853616_49853914 | RN7SL3 | 1094.1 |  |
| chr2_216871456_216871639 | LINC01921 | 1085.3 |  |
| chr22_20341524_20341548 | AC007731.1 | 1073.8 |  |
| chr5_7305074_7305203 | AC091951.1 | 1049.6 |  |
| chr1_152884403_152885047 | SMCP | 1045.0 | Sperm Mitochondria Associated Cysteine Rich Protein, Exon 2 |
| chr15_28846220_28846249 | GOLGA6L7 | 1041.0 |  |
| chr22_18424977_18425035 | FAM230A | 980.6 |  |
| chr2_88788442_88788514 | ANKRD36BP2 | 919.6 |  |
| chr22_18736559_18736583 | LINC01662 | 889.9 |  |
| chr5_7301753_7301894 | AC091951.1 | 867.6 |  |
| chr2_232816871_232817032 | GIGYF2 | 846.9 | GRB10 Interacting GYF Protein 2, Exon 21 |
| chr5_177729023_177729053 | FAM153A | 833.9 |  |
| chr16_14997748_14997826 | PDXDC1 | 815.9 |  |
| chr1_144560666_144560829 | RF00003 | 807.9 |  |
| chr14_60245752_60246046 | PPM1A | 754.7 |  |
| chrM_5761_5891 | MT-TC,MT-TY | 748.9 | Mitochondrial |
| chrX_103712236_103712360 | TMEM31 | 746.6 |  |
| chr2_232790698_232790915 | GIGYF2 | 732.6 | GRB10 Interacting GYF Protein 2, Exon 10 |

**Table S3. REs consistently altered in at least two MARS study visits.** REs shown to be differential solely due to batch effects were removed. Batch effect was defined as having a mean control expression of less than 1 RPKM. Genes with multiple affected REs are indicated in bold.

| Expression change | Element Identifier | Gene Symbol | RE class |
| --- | --- | --- | --- |
| Enriched in IBD | chr10_49703080_49703099 | C10orf53 | NOVEL_INTRONIC |
| Enriched in IBD | chr11_22674773_22675014 | GAS2 | EXON |
| Enriched in IBD | chr1_245677221_245677520 | KIF26B | NOVEL_INTRONIC |
| Enriched in IBD | chr15_33244895_33245438 | TMCO5B | EXON |
| Enriched in IBD | chr1_90497041_90497080 | NA | NOVEL_ORPHAN |
| Enriched in IBD | chr19_37551371_37551665 | ZNF571-AS1,ZNF540 | EXON |
| Enriched in IBD | chr2_178535732_178535828 | TTN-AS1 | EXON |
| Enriched in IBD | chr21_8259686_8259815 | RNA5-8S5 | NOVEL_10KB_EXON |
| Enriched in IBD | chr2_200797499_200797575 | AC007163.1 | EXON |
| Enriched in IBD | chr2_202346319_202346358 | AC064836.2 | NOVEL_10KB_EXON |
| Enriched in IBD | chr22_41174591_41174824 | EP300-AS1 | EXON |
| Enriched in IBD | chr2_30091379_30091408 | AC016907.2 | NOVEL_INTRONIC |
| Enriched in IBD | chr2_97149295_97149361 | **ANKRD36** | EXON |
| Enriched in IBD | chr2_97151879_97151939 | **ANKRD36** | EXON |
| Enriched in IBD | chr2_97183582_97183654 | **ANKRD36** | EXON |
| Enriched in IBD | chr3_113805354_113805525 | ATP6V1A | EXON |
| Enriched in IBD | chr3_47772817_47772936 | SMARCC1 | EXON |
| Enriched in IBD | chr3_52800965_52801146 | ITIH3 | EXON |
| Enriched in IBD | chr4_141231439_141231485 | ZNF330 | EXON |
| Enriched in IBD | chr4_15007305_15007595 | CPEB2 | EXON |
| Enriched in IBD | chr5_176586651_176586842 | **CDHR2** | EXON |
| Enriched in IBD | chr5_176589031_176589182 | **CDHR2** | EXON |
| Enriched in IBD | chr6_147001441_147001543 | STXBP5-AS1 | EXON |
| Enriched in IBD | chr9_34839387_34839416 | FAM205BP | NOVEL_10KB_EXON |
| Enriched in IBD | chrX_126731815_126731844 | MTCYBP38 | NOVEL_10KB_EXON |
| Enriched in IBD | chrY_12915883_12916027 | DDX3Y | EXON |
| Enriched in Control | chr15_29716611_29716838 | **TJP1** | EXON |
| Enriched in Control | chr15_29718266_29719138 | **TJP1** | EXON |
| Enriched in Control | chr19_10118668_10118727 | EIF3G | EXON |
| Enriched in Control | chr19_11024331_11024438 | SMARCA4 | EXON |
| Enriched in Control | chr20_38517784_38518000 | RALGAPB | EXON |
| Enriched in Control | chr20_410412_410764 | RBCK1 | EXON |
| Enriched in Control | chr20_62256057_62256221 | OSBPL2 | EXON |
| Enriched in Control | chr3_57268398_57268487 | APPL1 | EXON |
| Enriched in Control | chr4_42070526_42070691 | SLC30A9 | EXON |
| Enriched in Control | chr7_35872666_35872766 | SEPT7 | EXON |
| Enriched in Control | chr7_99353909_99354121 | **ARPC1A,AC004922.1** | EXON |
| Enriched in Control | chr7_99358340_99358415 | **ARPC1A,AC004922.1** | EXON |
| Enriched in Control | chr7_99359534_99359738 | **ARPC1A,AC004922.1** | EXON |
| Enriched in Control | chrM_14747_15953 | MT-CYB | EXON |

**Table S4. Number of REs altered by DBP.** The column “Study arm” indicates the MARS study arm of interest, while the column “Visit comparison” indicates the model comparison of interest. The column “RNA levels” indicates the observed change in RNA levels, while RE count enumerates the REs with the indicated expression change.

| Study arm | Visit comparison | RNA levels | RE count |
| --- | --- | --- | --- |
| **B_1_HB_2_** | B_1_ visit to H visit | Increase | 1021 |
|  | B_1_ visit to H visit | Decrease | 2630 |
|  | H visit to B_2_ visit | Increase | 665 |
|  | H visit to B_2_ visit | Decrease | 666 |
| **H_1_BH_2_** | H_1_ visit to B visit | Increase | 1150 |
|  | H_1_ visit to B visit | Decrease | 293 |
|  | B visit to H_2_ visit | Increase | 832 |
|  | B visit to H_2_ visit | Decrease | 779 |

**Table S5. DBP-altered exonic REs overlapping genes associated with sperm motility.** All murine genes in MGI database with the associated Gene Ontology term “sperm motility” (GO:0097722, <http://www.informatics.jax.org/go/term/GO:0097722>) were downloaded and transformed into the HGNC (human) gene symbol using custom R code and the BiomaRt package. The gene symbols associated with the differential exonic REs, partitioned according to expression change, were then overlapped with the list of HGNC gene symbols. All displayed gene names represent the gene symbol overlaps.

| B_1_HB_2_ arm | | | | H_1_BH_2_ arm | | | |
| --- | --- | --- | --- | --- | --- | --- | --- |
| B_1_ to H transition;  Increased levels | B_1_ to H transition;  Decreased levels | H to B_2_ transition;  Increased levels | H to B_2_ transition;  Decreased levels | H_1_ to B transition;  Increased levels | H_1_ to B transition;  Decreased levels | B to H_2_ transition;  Increased levels | B to H_2_ transition;  Decreased levels |
| CATSPERD | ATP1A4 | WDR66 | CATSPERD | CELF3 | IFT88 | IFT88 | TEKT5 |
| TTLL1 | WDR66 | GAPDHS | DNAH1 | ATP1A4 |  | GAPDHS | UBE2B |
|  | TEKT2 | IQCF1 |  |  |  | DNAI1 |  |
|  | TEKT5 |  |  |  |  |  |  |
|  | DRC7 |  |  |  |  |  |  |
|  | CFAP44 |  |  |  |  |  |  |
|  | DDX4 |  |  |  |  |  |  |
|  | DNAJA1 |  |  |  |  |  |  |

**Table S6. P-values of Gene ontology enrichment from differential MARS REs**. The gene names associated with differential Exonic, novel Near-exon, or novel Intronic REs were compiled and used as input to Genomatix’s GeneRanker function. The signaling pathways and literature-based pathways were summarized and presented based on common themes. The raw and adjusted p-values, in that order, are presented after each ontological term.

|  | Acute change | Recovery |
| --- | --- | --- |
| **Study arm** | *Upregulated REs* 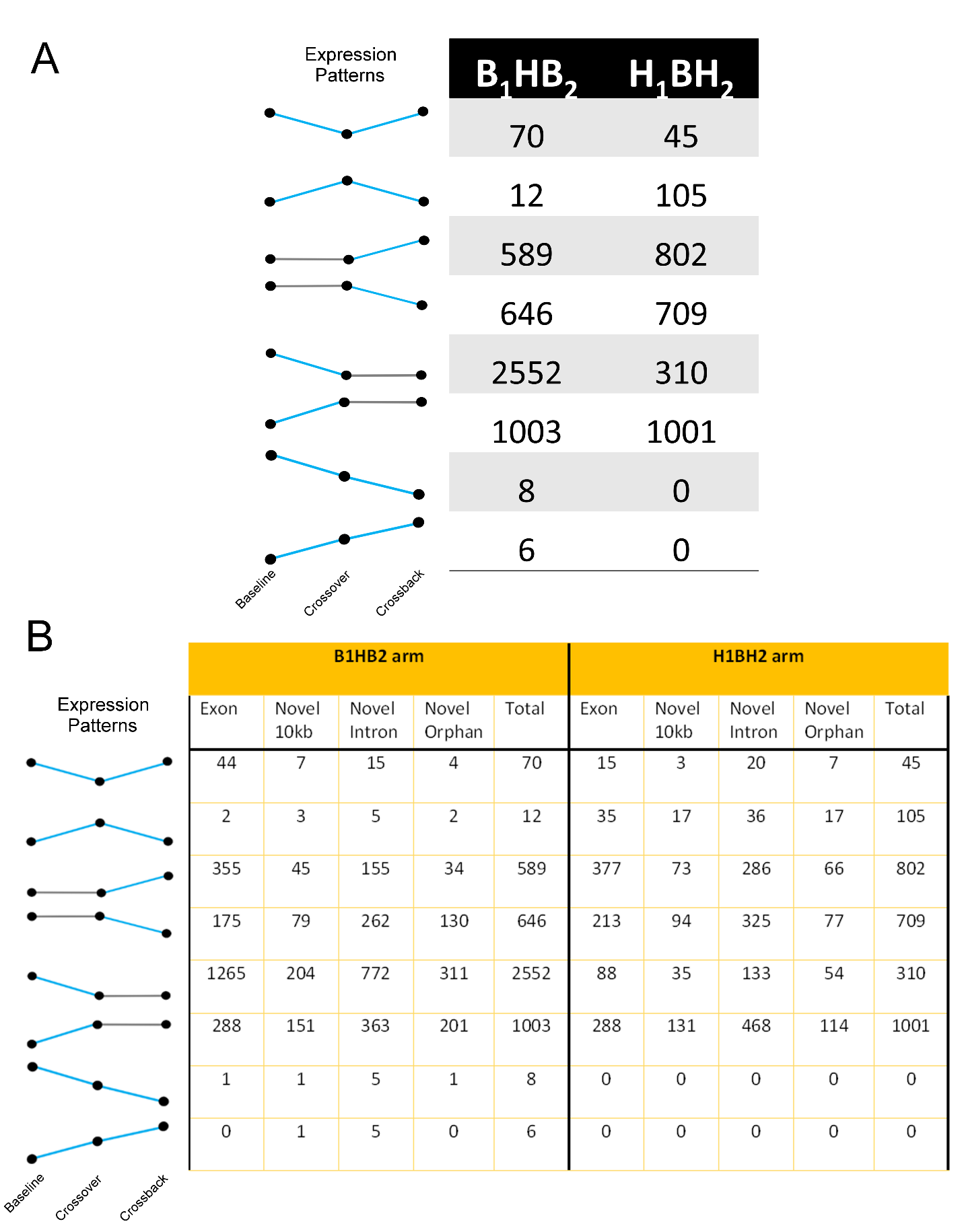 | *Upregulated REs* 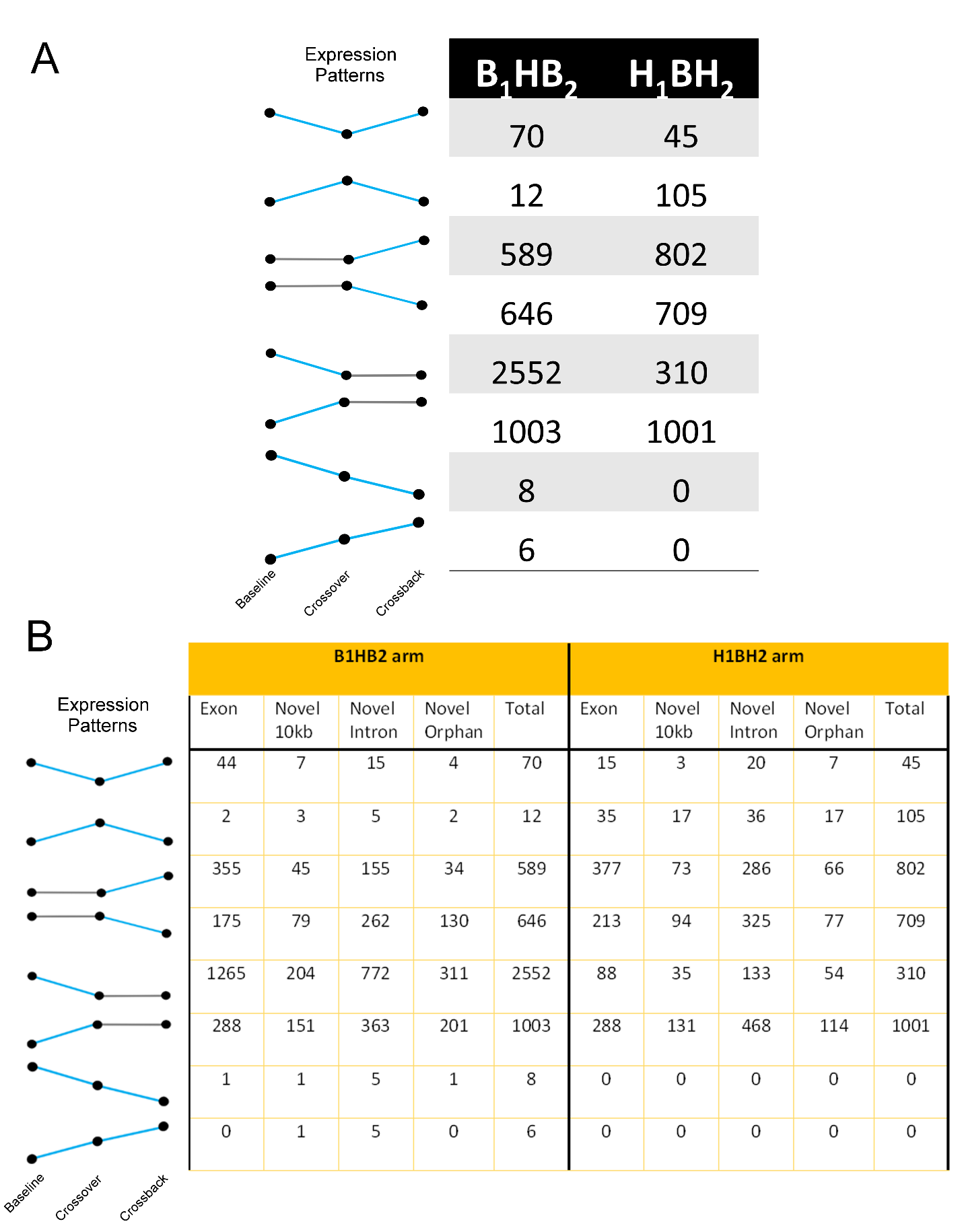 |
| **B_1_HB_2_** | Amino acid metabolism (1.0e-6, 0e0) | NGF signaling (5.4e-4, 0e0) |
|  | TNF-alpha (3.4e-4, 0e0) | EGFR signaling (1.5e-3, 0e0) |
|  | 14-3-3 protein signaling (4.0e-4, 1.0e-3) | RAN signaling (1.9e-3, 3.0e-3) |
|  |  |  |
|  | *Downregulated REs* 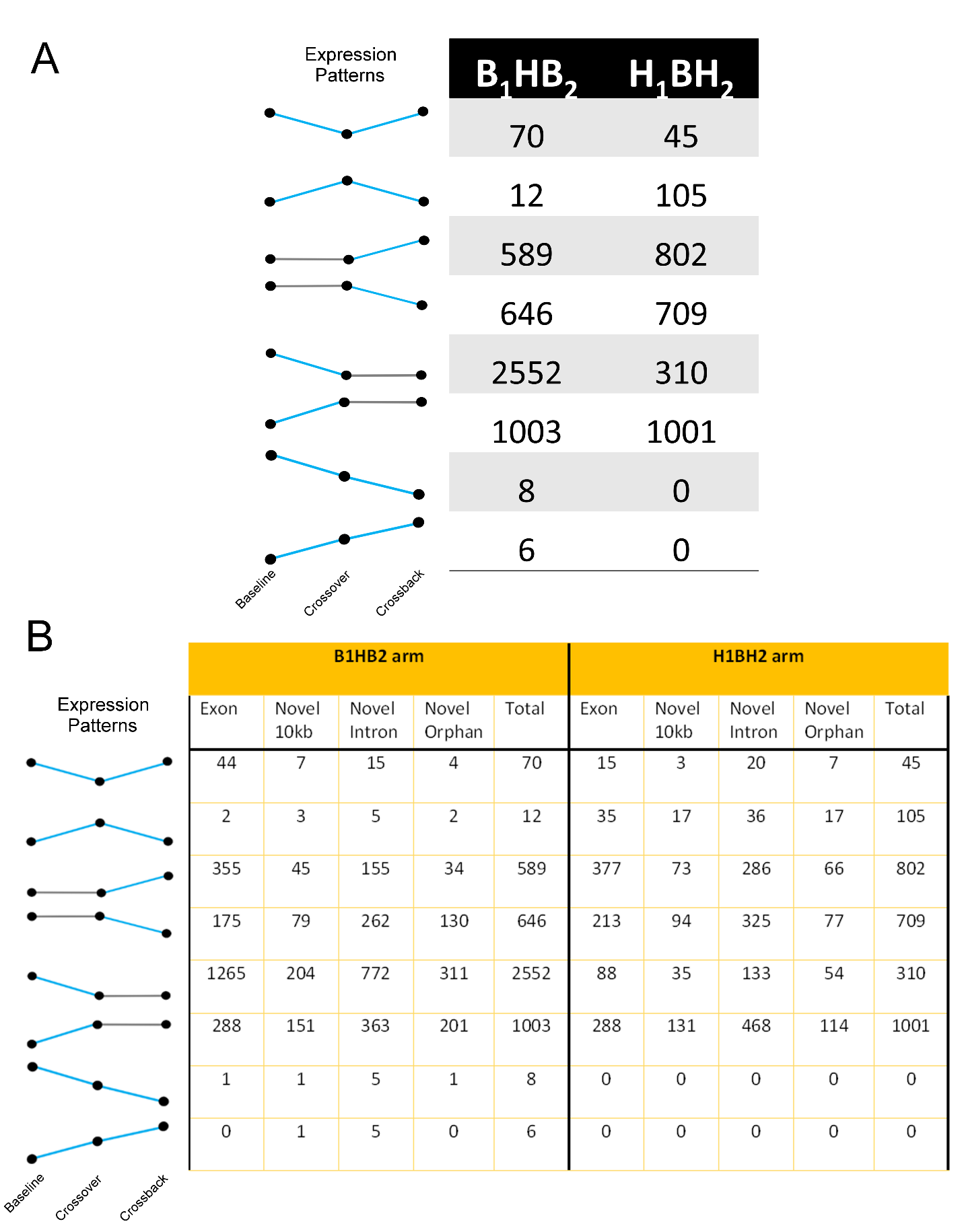 | *Downregulated REs* 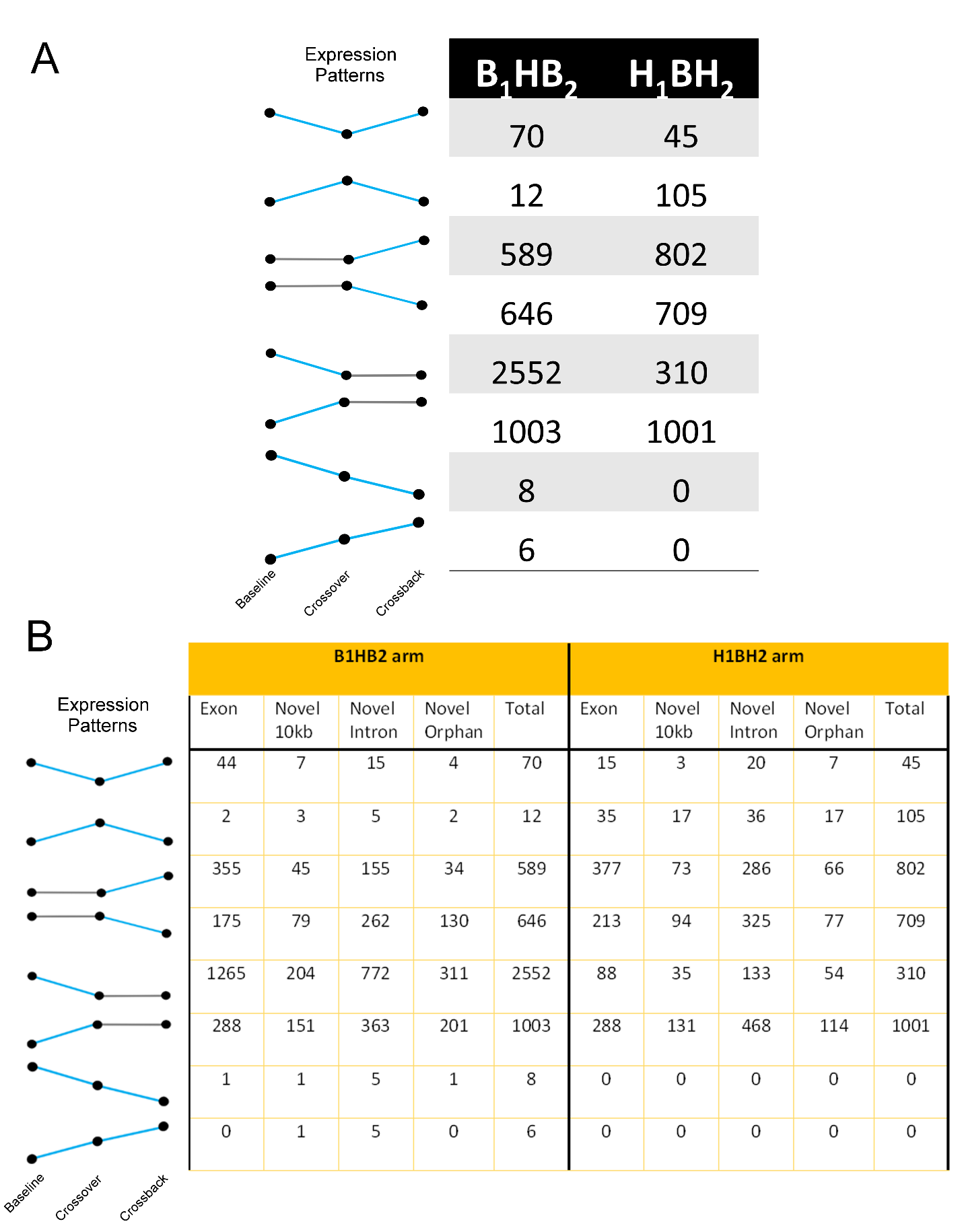 |
|  | RAN cycling (3.1e-6, 0e0) | NGF signaling (1.7e-4, 0e0) |
|  | Focal adhesion kinase signaling  (4.7e-4, 0e0) | EGFR signaling (4.8e-4, 0e0) |
|  | Ras GTPase binding (3.8e-9, 0e0) | Protein Kinase D and N (3.2e-5, 0e0) |
|  |  |  |
|  |  |  |
| **H_1_BH_2_** | *Upregulated REs* 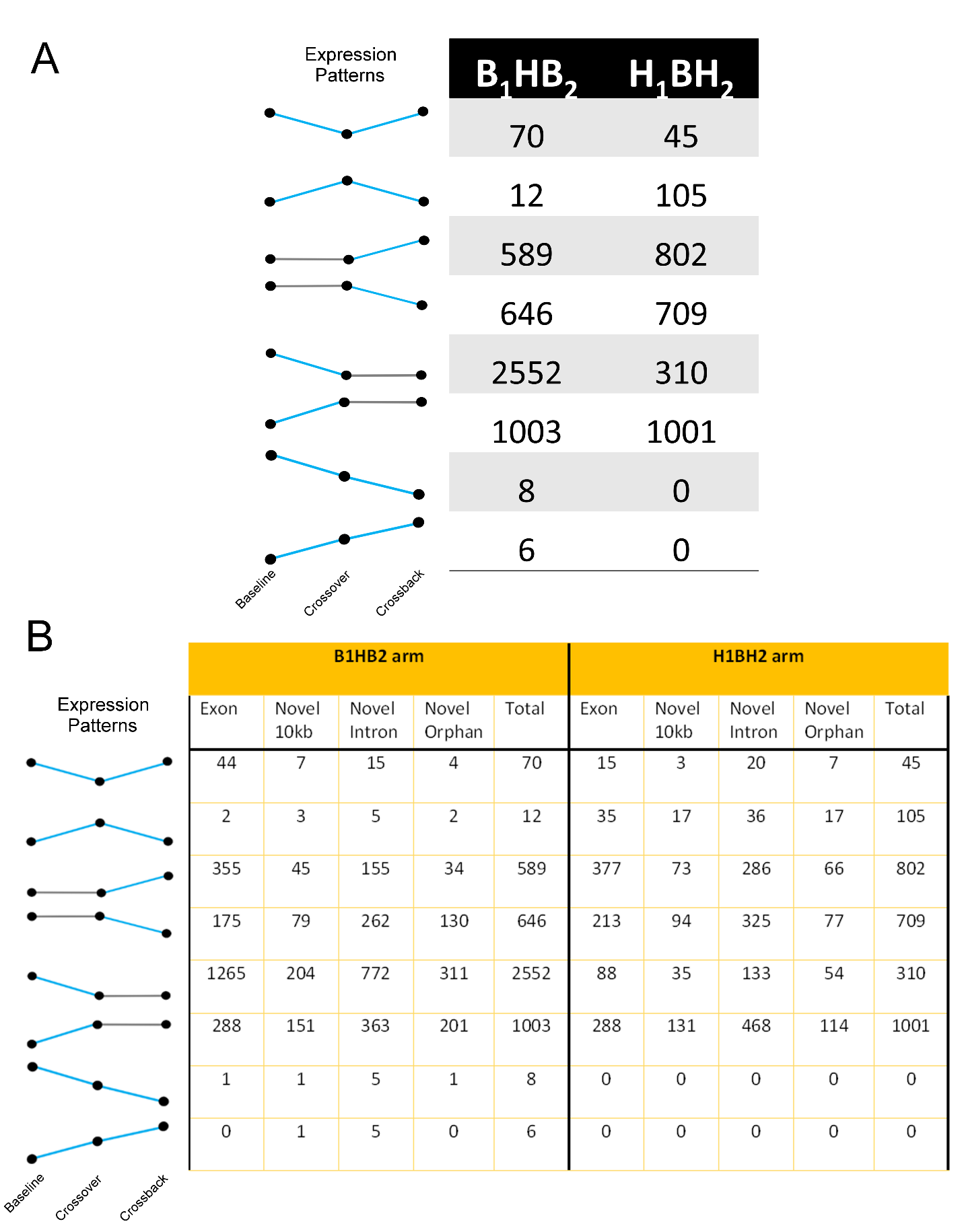 | *Upregulated REs* 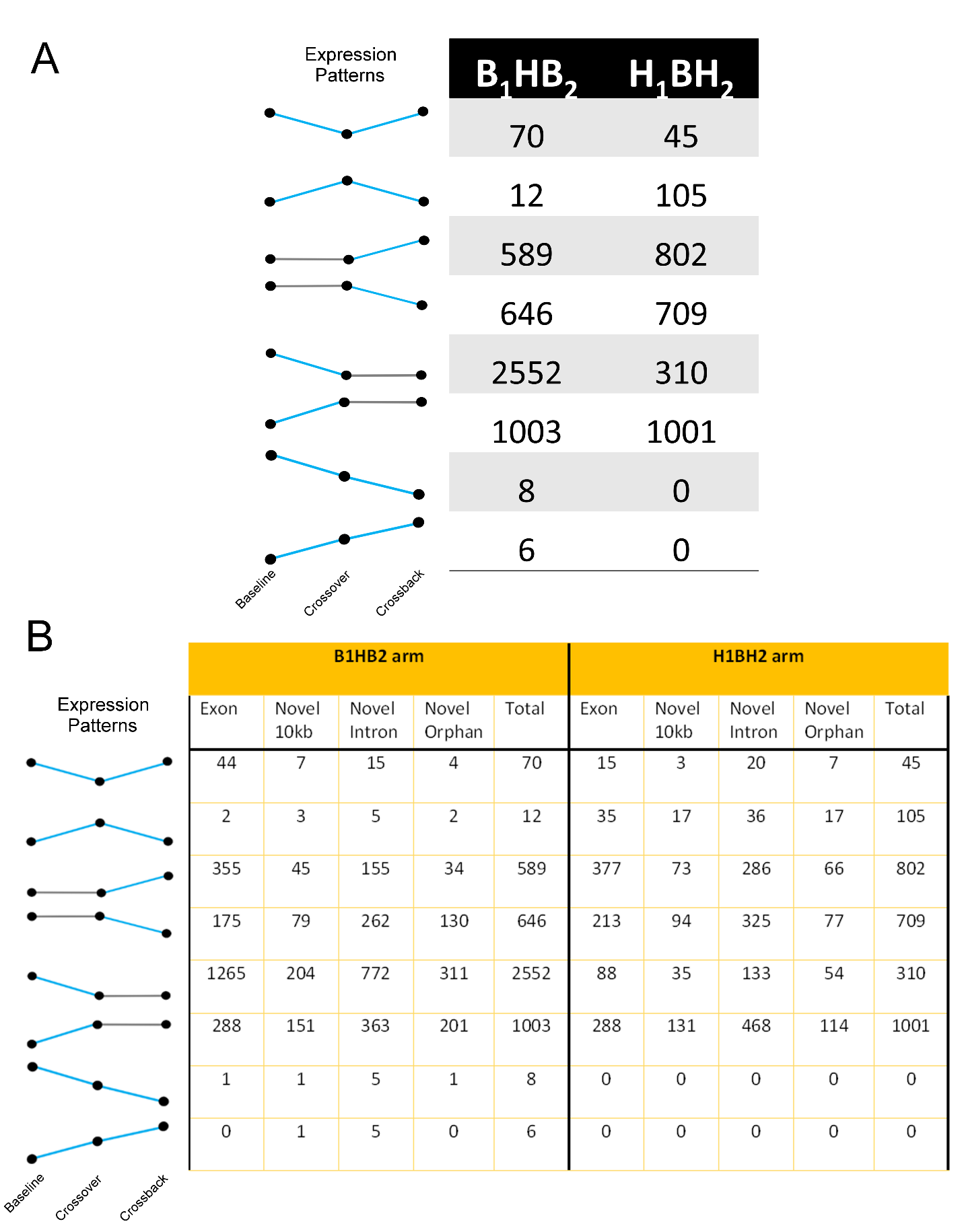 |
|  | Arf6 trafficking (1.7e-3, 3.0e-3) | EGFR1 (1.9e-3, 3.0e-3) |
|  | EphrinB-EPHB pathway (3.6e-4, 1e-3) | Calmodulin (5.7e-4, 1.0e-3) |
|  | Cytoskeleton organization (2.0e-7, 0e0) | Organelle biogenesis and maintenance (8.5e-4, 4.0e-3) |
|  |  |  |
|  | *Downregulated REs* 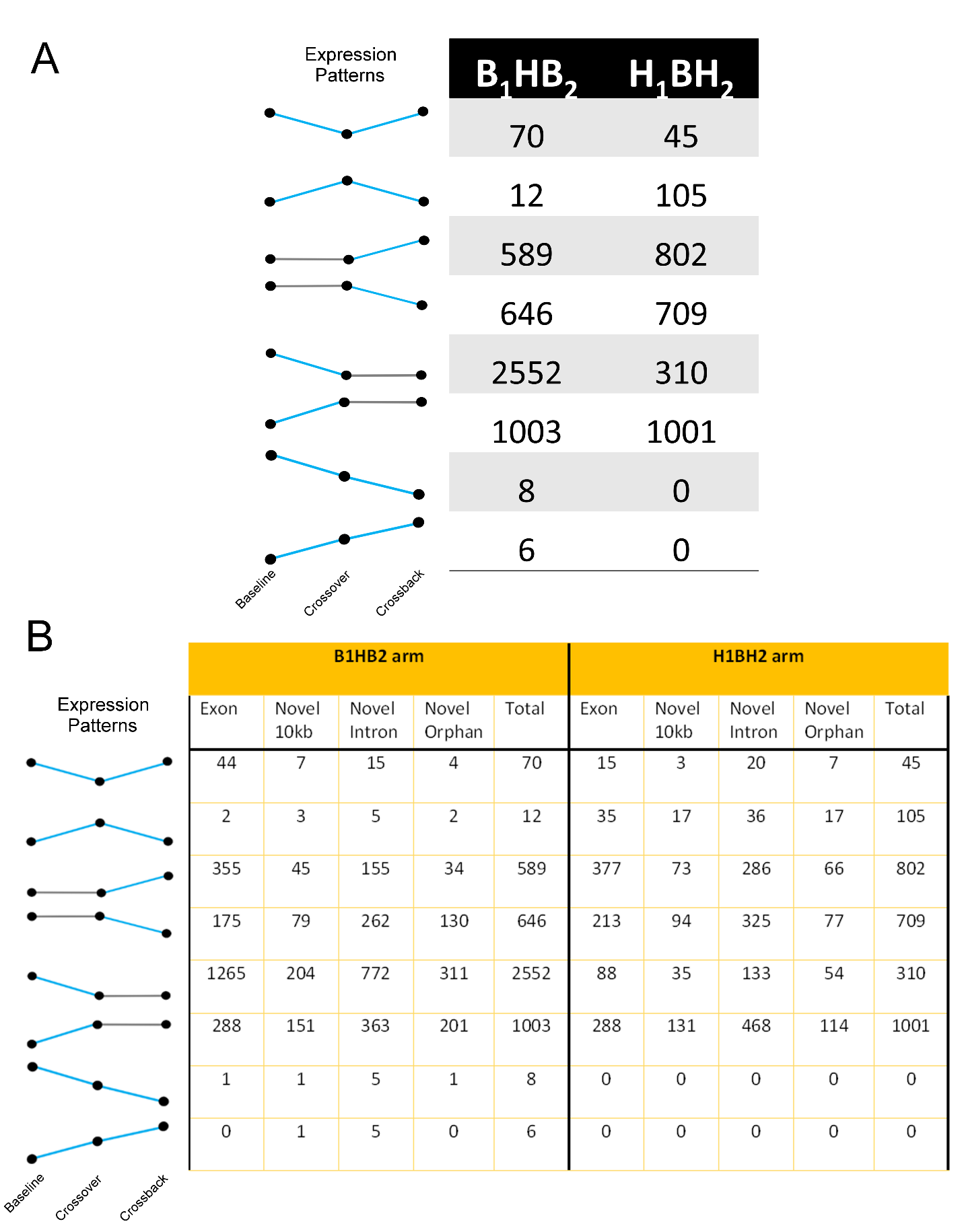 | *Downregulated REs* 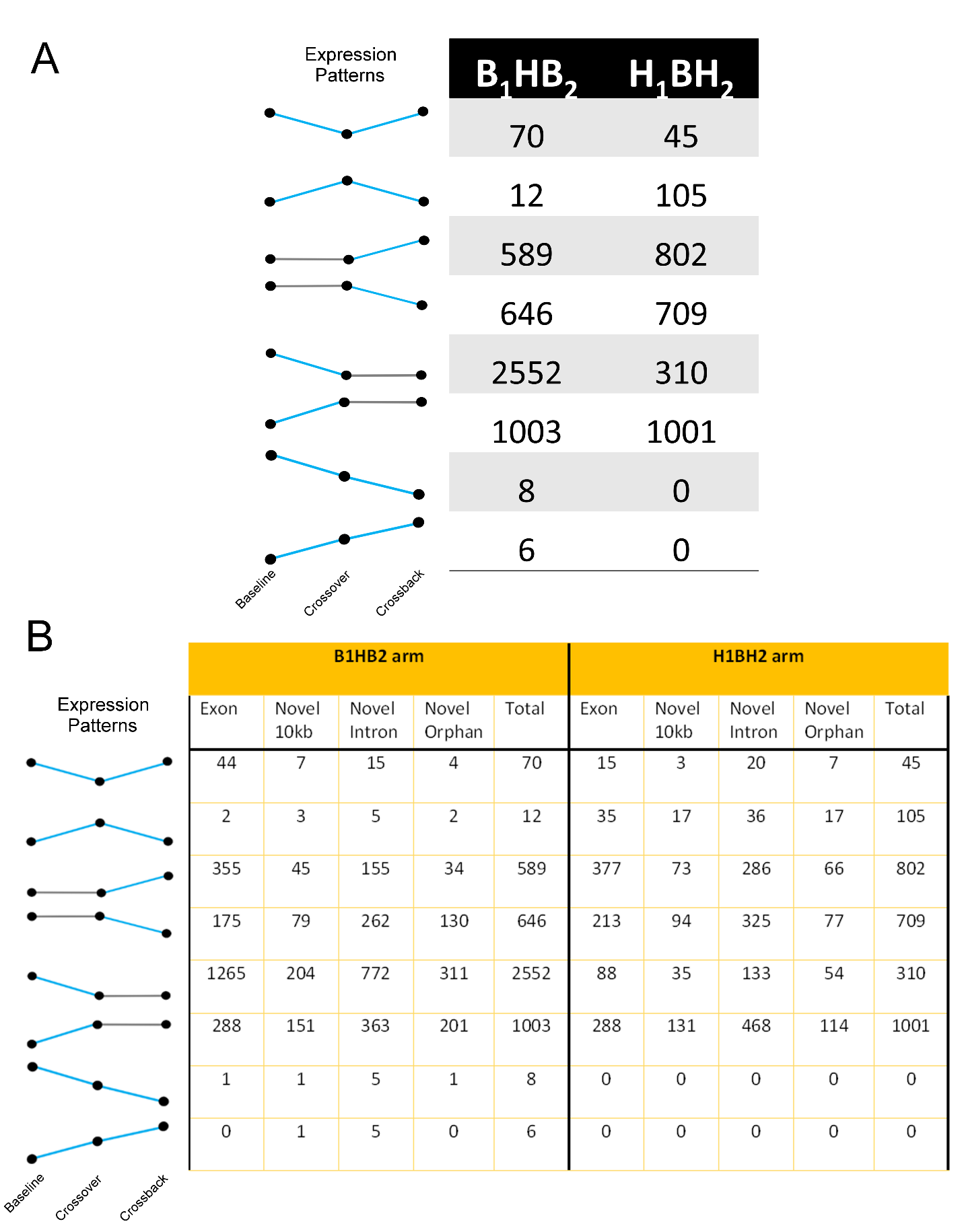 |
|  | ER to Golgi transport (9.8e-5, 1.0e-3) | Vesicle-mediated transport (2.3e-4, 0e0) |
|  | Axon guidance (5.4e-3, 4.0e-3) | Chromatin organization (1.3e-3, 0e0) |
|  | Organelle organization (1.5e-6, 0e0) | Coregulation of Androgen receptor activity (4.4e-3, 5.0e-3) |

**Table S7. Highly expressed small RNAs** in MARS small RNA libraries. “small RNA ID” indicates the type of small RNA, while the adjacent “Median RPM” column indicates the median expression value in RPM, across the MARS small RNA libraries.

| Small RNA ID | Median RPM | Small RNA ID | Median RPM | Small RNA ID | Median RPM |
| --- | --- | --- | --- | --- | --- |
| LSU-rRNA_Hsa | 432015.7 | hsa_piR_011482 | 277.5 | hsa_piR_022373 | 92.7 |
| SSU-rRNA_Hsa | 109860.6 | FAM | 266.4 | 6kbHsap | 91.3 |
| chrM | 79524.8 | hsa-miR-34c-5p | 251.8 | hsa_piR_000794 | 91.2 |
| hsa_piR_016735 | 20387.8 | hsa-miR-16-5p | 250.8 | hsa_piR_000753 | 90.6 |
| TRNA_GLY | 15131.7 | hsa_piR_020485 | 247.2 | ENSG00000206588\|ENST00000383861 | 88.7 |
| 5S | 11970.6 | (GGA)n | 239.0 | ENSG00000206596\|ENST00000383869 | 88.7 |
| LSU-rRNA_Cel | 11758.8 | hsa-miR-30d-5p | 236.4 | ENSG00000206652\|ENST00000383925 | 88.7 |
| SSU-rRNA_Dme | 9003.4 | L1HS | 235.7 | ENSG00000206737\|ENST00000384010 | 88.7 |
| hsa_piR_000823 | 8419.9 | tRNA-Ala-GCA | 221.4 | ENSG00000207005\|ENST00000384278 | 88.7 |
| LSU-rRNA_Dme | 8057.4 | hsa-miR-92a-3p | 204.0 | ENSG00000207389\|ENST00000384659 | 88.7 |
| hsa_piR_000765 | 7829.1 | hsa-miR-335-5p | 203.0 | ENSG00000207513\|ENST00000384782 | 88.7 |
| Y4 | 6740.5 | hsa-miR-15b-5p | 202.4 | ENSG00000273768\|ENST00000615842 | 88.7 |
| hsa_piR_020326 | 6524.2 | hsa_piR_005032 | 196.8 | ENSG00000275405\|ENST00000619109 | 88.7 |
| tRNA-Asp-GAY | 5832.5 | hsa_piR_009628 | 195.0 | hsa-miR-25-3p | 87.1 |
| TRNA_GLU | 5684.4 | hsa_piR_017936 | 190.9 | hsa-miR-423-5p | 86.9 |
| HY1 | 3908.0 | tRNA-Ser-TCA_ | 186.4 | hsa_piR_020548 | 82.1 |
| tRNA-Leu-CTY | 3381.0 | hsa_piR_014620 | 184.1 | hsa_piR_018570 | 81.8 |
| 7SL | 3332.4 | L1 | 183.6 | HY3 | 81.2 |
| RRNA45 | 2521.4 | tRNA-Leu-CTG | 183.6 | hsa_piR_011480 | 79.2 |
| SSU-rRNA_Cel | 2508.6 | hsa-miR-34b-3p | 181.8 | hsa_piR_022316 | 79.2 |
| hsa_piR_004153 | 2288.6 | hsa-miR-122-5p | 181.1 | ENSG00000206585\|ENST00000383858 | 78.6 |
| LOR1I | 1575.5 | AmnSINE1_HS | 173.9 | SVA_B | 75.8 |
| TRNA_VAL | 1392.2 | hsa-miR-9-5p | 163.2 | tRNA-Gln-CAG | 75.5 |
| tRNA-Lys-AAG | 1229.5 | ENSG00000276027\|ENST00000362512 | 160.1 | HERVFH21I | 75.1 |
| ENSG00000283293\|ENST00000636484 | 1097.7 | hsa-miR-10b-5p | 156.2 | hsa-let-7f-5p | 74.9 |
| tRNA-Met | 1012.6 | hsa-miR-148a-3p | 155.4 | hsa_piR_008112 | 74.9 |
| tRNA-Met-i | 1012.6 | SVA_A | 155.2 | ENSG00000200156\|ENST00000363286 | 73.8 |
| hsa_piR_017716 | 940.3 | (C)n | 155.0 | SVA_F | 72.9 |
| tRNA-Ser-AGY | 933.3 | HERVH | 154.3 | hsa-miR-99a-5p | 72.8 |
| hsa_piR_019914 | 791.2 | hsa_piR_018148 | 152.1 | hsa-miR-30e-5p | 71.6 |
| hsa-let-7b-5p | 772.8 | tRNA-Ser-TCY | 151.6 | hsa_piR_004396 | 70.8 |
| tRNA-Leu-CTA_ | 738.7 | hsa-miR-125b-5p | 145.9 | hsa_piR_022444 | 69.7 |
| hsa_piR_008114 | 710.2 | hsa-let-7g-5p | 135.8 | hsa-let-7c-5p | 68.6 |
| hsa_piR_008113 | 677.8 | hsa_piR_001318 | 135.6 | SVA_D | 68.1 |
| hsa-miR-375 | 632.2 | hsa-miR-200c-3p | 135.2 | tRNA-Ser-TCA | 67.5 |
| tRNA-SeC(e)-TGA | 601.4 | hsa_piR_013517 | 132.6 | SVA_C | 66.9 |
| tRNA-Leu-CTA | 518.1 | hsa_piR_001312 | 125.3 | hsa_piR_008564 | 65.7 |
| tRNA-Val-GTA | 514.6 | hsa_piR_001184 | 120.5 | hsa_piR_005031 | 63.0 |
| hsa_piR_006046 | 459.4 | U1 | 117.2 | hsa-miR-29a-3p | 60.1 |
| hsa_piR_019825 | 457.5 | hsa-miR-10a-5p | 113.1 | hsa-miR-151a-5p | 59.3 |
| hsa-miR-21-5p | 428.2 | hsa-miR-320a | 111.2 | hsa_piR_020381 | 59.0 |
| U4B | 427.9 | hsa_piR_018147 | 108.8 | hsa-miR-28-5p | 56.6 |
| hsa-let-7a-5p | 416.5 | tRNA-Ile-ATT | 107.6 | hsa_piR_019752 | 56.4 |
| tRNA-Pro-CCA | 381.7 | hsa_piR_013516 | 105.4 | SVA_E | 56.3 |
| hsa-miR-30a-5p | 324.1 | tRNA-Ala-GCY_ | 103.9 | hsa_piR_000805 | 56.2 |
| hsa-miR-191-5p | 320.2 | hsa_piR_017845 | 102.7 | hsa-miR-93-5p | 55.8 |
| hsa-miR-26a-5p | 309.7 | hsa-miR-30c-5p | 102.5 | ENSG00000274574\|ENST00000614774 | 55.0 |
| hsa_piR_015249 | 302.7 | HERV3 | 102.2 | ENSG00000277918\|ENST00000610976 | 55.0 |
| tRNA-Leu-TTG | 291.3 | hsa_piR_007373 | 96.8 | hsa_piR_003032 | 54.7 |
| hsa_piR_009294 | 288.0 | L1PREC1 | 96.6 | hsa-miR-23a-3p | 54.3 |
| hsa_piR_020500 | 285.4 | hsa-miR-1323 | 95.9 | hsa-miR-125a-5p | 52.1 |
| tRNA-Pro-CCG | 278.4 | HY1_SINE | 94.0 | hsa-miR-200b-3p | 51.3 |

**Table S8. small RNAs altered by DBP.** Cells highlighted in lime green exhibit the same expression trend in both study arms, while those highlighted in light orange exhibit opposite expression trends.

| B1HB2 arm | | | H1BH2 arm | | |
| --- | --- | --- | --- | --- | --- |
| High DBP > Baseline DBP | | High DBP < Baseline DBP | High DBP > Baseline DBP | High DBP < Baseline DBP | High DBP < Baseline DBP |
| L1M2_5 | CHARLIE10 | | ENSG00000278274\|ENST00000384581 | ALRb | hsa_piR_016280 |
| AluYg6 | hsa_piR_016677 | | hsa-miR-10a-3p | AluSq | hsa_piR_016804 |
| CHARLIE3 | hsa_piR_016735 | | hsa-miR-29c-5p | AluSq10 | hsa_piR_017428 |
|  | hsa_piR_016742 | | hsa_piR_004834 | AluYf1 | hsa_piR_017591 |
|  | hsa_piR_019675 | | hsa_piR_020619 | AluYk12 | hsa_piR_017781 |
|  | hsa-miR-186-5p | |  | CHARLIE3 | hsa_piR_018717 |
|  | hsa-miR-192-5p | |  | ENSG00000222094\|ENST00000410162 | hsa_piR_018790 |
|  | hsa-miR-200a-3p | |  | ENSG00000253089\|ENST00000517280 | hsa_piR_018924 |
|  | hsa-miR-27b-3p | |  | (GAAA)n | hsa_piR_020497 |
|  | hsa-miR-499a-5p | |  | HERV-Fc1 | hsa_piR_021722 |
|  | MER54A | |  | hsa-miR-508-5p | hsa_piR_022016 |
|  | tRNA-Gln-CAA | |  | hsa_piR_000753 | hsa_piR_022107 |
|  |  | |  | hsa_piR_001809 | hsa_piR_022629 |
|  |  | |  | hsa_piR_003180 | hsa_piR_023415 |
|  |  | |  | hsa_piR_003220 | L1M2A_5 |
|  |  | |  | hsa_piR_003222 | L1M3A_5 |
|  |  | |  | hsa_piR_003538 | L1MC3 |
|  |  | |  | hsa_piR_004427 | LTR16A |
|  |  | |  | hsa_piR_005278 | LTR1F1 |
|  |  | |  | hsa_piR_005675 | LTR30 |
|  |  | |  | hsa_piR_005767 | LTR66 |
|  |  | |  | hsa_piR_006426 | MER41G |
|  |  | |  | hsa_piR_008114 | MER54 |
|  |  | |  | hsa_piR_008397 | MER61I |
|  |  | |  | hsa_piR_009228 | MER66C |
|  |  | |  | hsa_piR_012388 | MER72 |
|  |  | |  | hsa_piR_013247 | MLT1D |
|  |  | |  | hsa_piR_014317 | SVA_A |
|  |  | |  | hsa_piR_015103 | tRNA-Ile-ATT |
|  |  | |  | hsa_piR_015482 |  |
